# Reactive Oxygen Species in Breast Cancer: From Redox Dysregulation to ROS‐Responsive Therapeutics

**DOI:** 10.1002/cbf.70301

**Published:** 2026-09-04

**Authors:** Marija Nikolovska, Radoslav Stojchevski, Aleksandar Eftimov, Mitko Mladenov, Dimiter Avtanski, Nikola Hadzi‐Petrushev

**Affiliations:** ^1^ Faculty of Natural Sciences and Mathematics, Institute of Biology Ss. Cyril and Methodius University Skopje Macedonia; ^2^ Faculty of Medicine, Institute of Pathology Ss. Cyril and Methodius University Skopje Macedonia; ^3^ Friedman Diabetes Institute, Lenox Hill Hospital, Northwell Health New York New York USA; ^4^ Donald and Barbara Zucker School of Medicine at Hofstra/Northwell Hempstead New York USA; ^5^ Institute of Molecular Medicine, Feinstein Institutes for Medical Research Manhasset New York USA; ^6^ Institute of Bioelectronic Medicine, Feinstein Institutes for Medical Research Manhasset New York USA

**Keywords:** antioxidants, breast cancer, multidrug resistance, Nrf2, oxidative stress, reactive oxygen species (ROS), ROS‐responsive nanoagents, therapeutic strategies, tumor microenvironment

## Abstract

Breast cancer is the most frequently diagnosed malignancy among women worldwide, with 2.3 million new cases and approximately 670,000 deaths reported in 2022 alone. Despite advances in therapy, metastasis and acquired drug resistance remain major clinical challenges. Reactive oxygen species (ROS) play a dual role in breast cancer biology: physiological levels sustain normal cellular signaling, moderately elevated levels promote tumorigenesis through DNA damage, proto‐oncogene activation, and tumor suppressor inactivation, while excessive accumulation can trigger cancer cell death. This review examines how redox dysregulation contributes to breast cancer initiation and progression through key signaling pathways, including phosphoinositide 3‐kinase/protein kinase B (PI3K/AKT), mitogen‐activated protein kinase (MAPK), and Kelch‐like ECH‐associated protein 1–nuclear factor erythroid 2‐related factor 2 (Keap1‐Nrf2), as well as apoptotic cascades. We evaluate the evidence for dietary and synthetic antioxidants—melatonin, curcumin, vitamins C and E, and carotenoids—as chemopreventive and adjuvant agents, highlighting both their therapeutic promise and the conflicting data on their safety during cancer treatment. We further discuss emerging ROS‐responsive nanoagents for targeted drug delivery and immunotherapy, and strategies to exploit redox vulnerabilities in multidrug‐resistant breast cancer cells, including induction of ferroptosis, an iron‐dependent cell death pathway driven by lipid peroxide accumulation that has emerged as a promising vulnerability in therapy‐resistant and mesenchymal‐phenotype tumors. Recent advances in machine learning and multi‐omics integration, which have begun to identify redox‐related gene signatures with prognostic and immunotherapy‐predictive value, further point toward precision redox oncology as an emerging clinically actionable framework. By integrating molecular mechanisms with translational advances, this review identifies current gaps and future directions for ROS‐targeted therapeutic strategies in breast cancer.

## Significance Statement

Breast cancer remains one of the leading causes of cancer‐related death in women, and drug resistance continues to limit treatment success. This review explains how reactive oxygen species act as double‐edged swords in breast cancer. At low levels, they fuel tumor growth and survival; at high levels, they kill cancer cells. Understanding where this tipping point lies is key to designing smarter therapies. We synthesize how cancer cells exploit redox imbalance to resist chemotherapy and evade immune attack, and we highlight emerging strategies, including targeted nanomedicines, ferroptosis inducers, and AI‐driven redox profiling, that harness this vulnerability to improve patient outcomes.

**Abbreviations**
^1^O_2_
: singlet oxygen
5hmC: 5‐hydroxymethylcytosine
5mC: 5‐methylcytosine
8‐oxo‐dG: 8‐oxo‐2′‐deoxyguanosine
AFMK: N1‐acetyl‐N2‐formyl‐5‐methoxykynuramine
AI: artificial intelligence
AIARS: Artificial Intelligence‐aided Redox Signature
AKT: protein kinase B
AMK: N1‐acetyl‐5‐methoxykynuramine
AP‐1: activator protein‐1
AQP3: aquaporin 3
AQP8: aquaporin 8
ARE: antioxidant response element
ATBC: Alpha‐Tocopherol Beta Carotene Cancer Prevention Study
ATP: adenosine triphosphate
BACH1: BTB domain and CNC homolog 1
Bax: Bcl‐2‐associated X protein
Bcl‐2: B‐cell lymphoma 2
Bcl‐xL: B‐cell lymphoma‐extra large
BER: Base excision repair
BLIS: basal‐like and immune‐suppressed
BRCA1: breast cancer susceptibility gene 1
BRD4: bromodomain‐containing protein 4
cAMP: cyclic adenosine monophosphate
CARET: Beta‐Carotene and Retinol Efficacy Trial
CAT: catalase
CBP: CREB‐binding protein
CCL26: C‐C motif chemokine ligand 26
COX‐2: cyclooxygenase‐2
CPT: camptothecin
Cul3: Cullin 3
CYP: cytochrome P450
DAMPs: damage‐associated molecular patterns
DLG: Asp‐Leu‐Gly motif
DNBS: 2,4‐dinitrobenzenesulfonate
DOX: doxorubicin
EGFR: epidermal growth factor receptor
EMT: epithelial‐mesenchymal transition
ENTPD2: ectonucleoside triphosphate diphosphohydrolase 2
ER: estrogen receptor
ERK: extracellular signal‐regulated kinase
ERα: estrogen receptor alpha
ETC: electron transport chain
ETGE: Glu‐Thr‐Gly‐Glu motif
FLIP: FLICE‐like inhibitory protein
FUDR: 5‐floxuridine
GCLC: glutamate‐cysteine ligase catalytic subunit
GCLM: glutamate‐cysteine ligase modifier subunit
GPx: glutathione peroxidase
GPX4: glutathione peroxidase 4
GR: glutathione reductase
GSH: glutathione
GSSG: glutathione disulfide
GST: glutathione S‐transferase
G‐CSF: granulocyte colony‐stimulating factor
H_2_O_2_
: hydrogen peroxide
HA: hyaluronic acid
HAase: hyaluronidase
HDAC3: histone deacetylase 3
HER2: human epidermal growth factor receptor 2
HIF‐1α: hypoxia‐inducible factor 1‐alpha
HMGB1: high mobility group box 1
HO^−^1: heme oxygenase‐1
HR: Homologous recombination
IAP: inhibitor of apoptosis protein
ICD: immunogenic cell death
ICI: immune checkpoint inhibitor
IKK: IκB kinase
IL: interleukin
IV: intravenous
IκBα: nuclear factor of kappa light polypeptide gene enhancer in B‐cells inhibitor alpha
JNK: c‐Jun N‐terminal kinase
Keap1: Kelch‐like ECH‐associated protein 1
LAR: luminal androgen receptor
LASSO: least absolute shrinkage and selection operator
LOO•: lipid peroxyl radical
MAC: monocarbonyl analog of curcumin
MAPK: mitogen‐activated protein kinase
MDA: malondialdehyde
MDR: multidrug resistance
*MDR1*
: multidrug resistance protein 1 (gene)
MDSC: myeloid‐derived suppressor cell
MEK: mitogen‐activated protein kinase kinase
ML: machine learning
MMP: matrix metalloproteinase
MMR: Mismatch repair
MRP: multidrug resistance‐associated protein
MT1/MT2: melatonin receptors 1 and 2
mTOR: mammalian target of rapamycin
NAC: N‐acetylcysteine
NADPH: nicotinamide adenine dinucleotide phosphate (reduced)
NER: Nucleotide excision repair
*NFE2L2*
: nuclear factor erythroid 2‐like 2 (gene encoding Nrf2)
NF‐κB: nuclear factor kappa‐light‐chain‐enhancer of activated B cells
NHEJ: Non‐homologous end joining
NK: natural killer (cell)
NOX: NADPH oxidase
NQO1: NAD(P)H:quinone oxidoreductase 1
Nrf2: nuclear factor erythroid 2‐related factor 2
O_2_•^−^
: superoxide anion radical
ONOO^−^
: peroxynitrite
OSRG: oxidative stress‐related gene
p21: cyclin‐dependent kinase inhibitor 1A
p27: cyclin‐dependent kinase inhibitor 1B
p38: p38 mitogen‐activated protein kinase
p53: tumor protein p53
PARP: poly(ADP‐ribose) polymerase
PD‐1: programmed cell death protein 1
PD‐L1: programmed death‐ligand 1
PEITC: phenethyl isothiocyanate
PET: photoinduced electron transfer
PHD: prolyl hydroxylase domain
PI3K: phosphoinositide 3‐kinase
PRDX4: peroxiredoxin 4
PTEN: phosphatase and tensin homolog
PTM: post‐translational modification
P‐gp: P‐glycoprotein
Rbx1: RING‐box protein 1
RCT: randomized controlled trial
ROS: reactive oxygen species
SELECT: Selenium and Vitamin E Cancer Prevention Trial
siRNA: small interfering RNA
SLN: solid lipid nanoparticle
SOD: superoxide dismutase
STAT3: signal transducer and activator of transcription 3
TAC: total antioxidant capacity
TAM: tumor‐associated macrophage
TET: ten‐eleven translocation (dioxygenase)
TGF‐β: transforming growth factor beta
TK: thioketal
TME: tumor microenvironment
TNBC: triple‐negative breast cancer
TNF‐α: tumor necrosis factor alpha
Treg: regulatory T cell
TrxR: thioredoxin reductase
VEGF: vascular endothelial growth factor
•OH: hydroxyl radical

## 1 Introduction

Breast cancer is a highly heterogeneous disease, encompassing diverse subtypes that differ in molecular profiles, clinical behaviors, and responses to treatment [1, 2]. While certain malignancies are defined by pathognomonic molecular aberrations, breast cancer lacks a single unifying genetic driver, which complicates both classification and targeted therapy. The incidence of breast cancer in women increased worldwide between 2000 and 2018, from 1.05 million to 2.09 million, although the mortality rate has decreased [3, 4]. The number of cases is projected to increase by over 40% by 2040, to about 3 million cases each year due to population growth and aging. Similarly, deaths from breast cancer will increase by over 50%, to about 1 million in 2040 [5].

Accurate classification is crucial for optimizing patient management, monitoring, clinical trial selection, and translational research [2, 6]. Over time, breast cancer classification has progressively evolved from traditional morphology‐based systems to a more comprehensive approach that integrates tumor biomarkers, gene expression profiling, and genomic alterations [7, 8].

Among the recognized molecular subtypes, triple‐negative breast cancer (TNBC), which lacks estrogen receptor (ER), progesterone receptor (PR), and human epidermal growth factor receptor 2 (HER2) expression, is particularly associated with elevated basal oxidative stress and limited therapeutic options [9, 10]. Luminal and HER2‐enriched subtypes also exhibit redox alterations that influence treatment response and contribute to resistance mechanisms [11, 12]. This subtype‐dependent variation in redox biology highlights the importance of understanding reactive oxygen species (ROS)‐related processes in the context of breast cancer heterogeneity, which has direct implications for the therapeutic strategies discussed in this review. TNBC/basal‐like cell lines exhibit significantly higher baseline ROS levels than luminal (MCF‐7) or non‐tumorigenic (MCF10A) cells, as measured by dihydroethidium staining, and this elevated oxidative state cannot be further increased by exogenous H_2_O_2_, suggesting that TNBC cells operate near their maximal oxidative capacity [10]. Paradoxically, ER‐positive breast cancers show higher levels of 8‐hydroxydeoxyguanosine (8‐OHdG), a marker of oxidative DNA damage, than ER‐negative tumors, likely reflecting estrogen‐induced mitochondrial ROS generation through oxidative estrogen metabolism [13]. Transcriptomic ROS scoring across large patient cohorts (METABRIC, TCGA) has confirmed that high ROS pathway activity is significantly associated with worse overall survival in ER‐positive/HER2‐negative breast cancer but not in TNBC or HER2‐positive subtypes, suggesting that the prognostic significance of ROS is subtype‐dependent [14].

These differences extend to antioxidant defense architecture: glutathione peroxidase 4 (GPX4) expression is lowest in basal‐like tumors and highest in luminal subtypes, while nuclear factor erythroid 2‐related factor 2 (Nrf2) upregulation in TNBC contributes to both proliferation and chemoresistance through mechanisms distinct from those operating in ER‐positive disease [15]. The therapeutic consequences are substantial. Within TNBC itself, ferroptosis sensitivity varies markedly across transcriptomic subtypes: the luminal androgen receptor (LAR) subtype is hypersensitive to GPX4 inhibitors, whereas basal‐like and immune‐suppressed (BLIS) subtypes are comparatively resistant, and the combination of GPX4 inhibition with anti‐programmed cell death protein 1 (anti‐PD1) immunotherapy showed greater efficacy than either monotherapy in LAR models [16]. These observations collectively argue against a one‐size‐fits‐all approach to ROS‐targeted therapy in breast cancer and support subtype‐stratified redox interventions, a theme that recurs throughout Sections 4, 5, 6, 7.

The progression of this disease is predominantly influenced by genetic mutations, with oxidative stress being a key factor in all stages of carcinogenic development [17, 18]. Oxidative stress occurs due to an imbalance between free radical production and antioxidants, where ROS represent one of the most significant classes of oxidative mediators [18, 19]. The regulation of oxidative stress emerges as a key factor in both tumor development and the efficacy of anticancer treatment [12, 17]. Compared to normal cells, cancer cells often exhibit altered redox status, with numerous signaling pathways implicated in tumorigenesis directly or indirectly modulating ROS levels through metabolic mechanisms that drive tumor progression [12, 20]. The sustained elevation of ROS can alter metabolic pathways, promote epithelial‐mesenchymal transition, and facilitate metastasis [21, 22]. Many breast cancer patients routinely incorporate antioxidant supplements into their regimens during chemotherapy, radiation therapy, or hormone therapy [23]. Although the involvement of oxidative stress in cancer development and progression is well‐established, the precise molecular mechanisms underlying tumor initiation, metastatic advancement, and treatment responses remain incompletely understood. A more comprehensive understanding of how oxidative stress shapes the tumor microenvironment (TME) may reveal novel therapeutic strategies for breast cancer.

This narrative review synthesizes current evidence on the roles of ROS in breast cancer biology, with emphasis on redox dysregulation mechanisms, antioxidant interventions, immunotherapy interactions, and strategies to overcome multidrug resistance (MDR). Literature was identified through systematic searches of PubMed/MEDLINE, Scopus, and Web of Science databases using combinations of keywords including “reactive oxygen species,” “oxidative stress,” “breast cancer,” “redox signaling,” “antioxidant,” “drug resistance,” and “immunotherapy,” with priority given to peer‐reviewed original research articles, clinical trials, and authoritative reviews published through 2024–2025. Reference lists of key articles were additionally screened to ensure comprehensive coverage.

## 2 ROS

ROS encompass a diverse group of small molecules produced by the body, including radicals and non‐radical species. Radicals such as superoxide (O_2_^−^•), hydroxyl (OH•), and peroxyl (RO_2_•) are short‐lived, highly electrophilic, and reactive molecules with an unpaired electron in their outer shell. Non‐radical species include hypochlorous acid (HOCl), ozone (O_3_), singlet oxygen (^1^O_2_), and hydrogen peroxide (H_2_O_2_) [24, 25]. ROS formation typically begins with a one‐electron reduction of molecular oxygen to form the superoxide anion, which can convert to H_2_O_2_ either spontaneously or through superoxide dismutase activity. Further reactions in the cascade of ROS production form peroxynitrite (ONOO^−^) from superoxide with nitric oxide (NO), HOCl from H_2_O_2_ via peroxidase, or hydroxyl radical (•OH) through the iron‐catalyzed Fenton reaction [26, 27]. Notably, peroxynitrite is classified as a reactive nitrogen species (RNS), but its formation from superoxide links it closely to ROS‐mediated damage.

**Figure 1:**
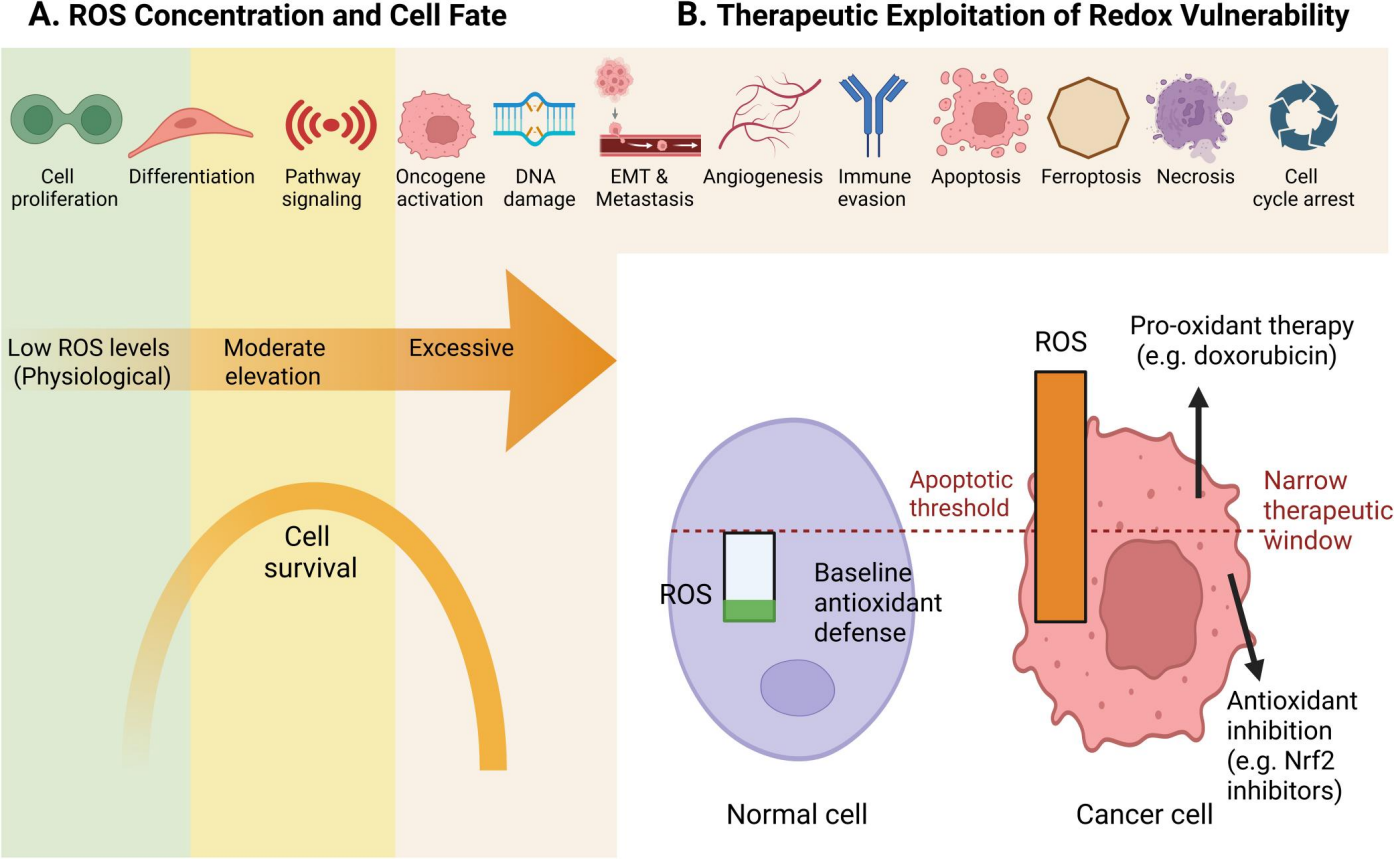
Biphasic effects of reactive oxygen species (ROS) on cell fate and therapeutic exploitation of redox vulnerability in breast cancer. (A) Physiological ROS levels support proliferation, differentiation, and signaling for normal homeostasis. Moderate elevations drive cancer hallmarks (oncogene activation, DNA damage, EMT, angiogenesis, and immune evasion). Excessive ROS overwhelm defenses, triggering oxidative stress and cell death (apoptosis, ferroptosis, necrosis) or cell cycle arrest. (B) Cancer cells exhibit elevated baseline ROS and a narrow therapeutic window, selectively exploitable by pro‐oxidant agents (e.g., doxorubicin and radiation) or antioxidant pathway inhibitors (e.g., Nrf2 inhibitors, buthionine sulfoximine). Created in BioRender. M. Nikolovska, 2026, https://BioRender.com/gpmoemk.

ROS serve as signaling molecules regulating physiological processes and cellular homeostasis. However, excessive ROS accumulation damages cellular organelles and processes, compromising normal physiological functions [28]. Imbalance between ROS production and cellular antioxidant defenses has been implicated in the pathogenesis of various diseases, including cancer [19, 28]. To counteract oxidative damage, cells employ both enzymatic and non‐enzymatic antioxidant defense systems. Enzymatic defenses include superoxide dismutase (SOD), which converts superoxide to H_2_O_2_; catalase, which decomposes H_2_O_2_ to water and oxygen; glutathione peroxidase (GPx), which reduces H_2_O_2_ and lipid hydroperoxides using glutathione (GSH) as a co‐substrate; and the thioredoxin/peroxiredoxin system. Non‐enzymatic antioxidants include GSH, vitamins C and E, and carotenoids, many of which are discussed in detail in subsequent sections [26, 28]. When these defenses are overwhelmed, the resulting oxidative stress impairs cellular function [19]. The biological outcome is therefore determined by ROS concentration: physiological levels support signaling and homeostasis, moderate elevations promote malignant transformation, and excessive accumulation triggers cell death [25] (Figure 1).

**Figure 2:**
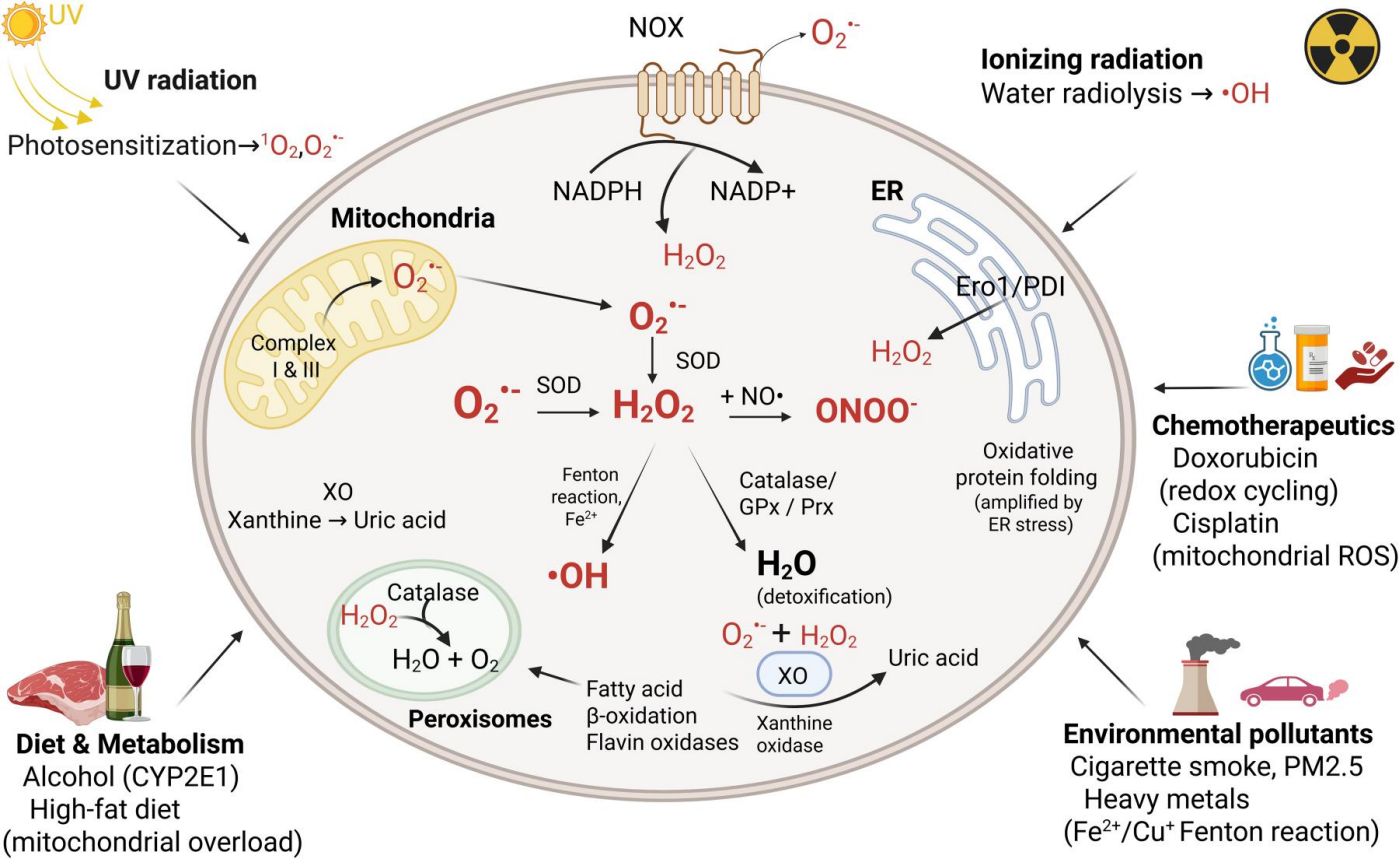
Endogenous and exogenous sources of reactive oxygen species (ROS) in breast cancer. Major endogenous generators include mitochondrial electron transport (Complex I/III), NADPH oxidases (NOX1–5, DUOX1–2), peroxisomal β‐oxidation, xanthine oxidase, and endoplasmic reticulum oxidative protein folding. Exogenous sources comprise UV and ionizing radiation, environmental pollutants (cigarette smoke, PM2.5, heavy metals), diet/metabolism (alcohol, high‐fat diet), and chemotherapeutics (doxorubicin, cisplatin). Primary ROS species and key conversion/detoxification routes are shown. Created in BioRender. M. Nikolovska, 2026, https://BioRender.com/8bzz74h.

ROS arise from endogenous or exogenous sources [28] (Figure 2). Endogenous sources include the NADPH oxidase (NOX), a membrane‐bound enzyme that transfers electrons from (nicotinamide adenine dinucleotide phosphate [NADPH]) to oxygen to form superoxide, of which seven human homologs exist: NOX1, NOX2, NOX3, NOX4, NOX5, DUOX1, and DUOX2 [29, 30]; the mitochondrial respiratory chain that generates superoxide during oxidative phosphorylation [27, 31]; the endoplasmic reticulum (ER) stress that produces H_2_O_2_ [32]; and peroxisomes that contain catalase and flavin oxidases that yield H_2_O_2_ [33]. Additionally, xanthine oxidase (XO) generates both O_2_^−^• and H_2_O_2_ during purine catabolism, converting hypoxanthine to xanthine and xanthine to uric acid. XO activity is elevated in several cancers and contributes to oxidative stress in the tumor microenvironment [34]. Of particular relevance to breast cancer, cytochrome P450 enzymes, notably CYP1A1, CYP1B1, and CYP3A4, catalyze the hydroxylation of estradiol to catechol estrogens (2‐hydroxy‐ and 4‐hydroxyestradiol), which can undergo further oxidation to semiquinone and quinone intermediates. These electrophilic metabolites undergo redox cycling, generating O_2_^−^• and H_2_O_2_, and can also form depurinating DNA adducts at adenine and guanine, directly linking estrogen metabolism to oxidative DNA damage and breast carcinogenesis [35, 36].

Exogenous factors also contribute to ROS formation. For example, ultraviolet (UV) radiation, particularly UVA and UVB, induces the generation of singlet oxygen, superoxide anion, and hydroxyl radicals through the excitation of endogenous chromophores and the initiation of lipid peroxidation cascades [37]. Ionizing radiation, including X‐rays and gamma rays, generates ROS primarily through radiolysis of water molecules in cells [38]. Other important exogenous inducers include environmental pollutants such as cigarette smoke, particulate matter (PM2.5), and heavy metals [39].

Dietary and lifestyle factors, such as alcohol, which generates ROS through CYP2E1‐mediated oxidative metabolism [40], as well as pharmaceutical agents including non‐steroidal anti‐inflammatory drugs (NSAIDs), chemotherapeutics (e.g., doxorubicin), and immunosuppressants, can also promote ROS generation through redox cycling or altered mitochondrial metabolism [41]. Aging is characterized by reduced antioxidant defenses and increased ROS, resulting in a shift toward oxidative stress that is further amplified in cancer [42]. Additionally, cellular redox balance affects inflammation, where activated immune cells produce elevated levels of proinflammatory cytokines and ROS, establishing a feed‐forward loop that promotes chronic inflammation and sustained DNA damage [43].

In addition to apoptosis and necrosis, ROS can trigger ferroptosis, an iron‐dependent form of regulated cell death driven by the accumulation of lipid peroxides. Therapy‐resistant cancer cells, particularly those with mesenchymal features, have been shown to be highly susceptible to ferroptosis, making it a subject of growing interest in breast cancer research [44].

## 3 Spatial and Temporal Compartmentalization of ROS

The biological outcome of ROS production depends not only on the overall concentration but also on the identity, subcellular localization, and kinetic properties of the specific species generated. Superoxide (O_2_^•−^), hydrogen peroxide (H_2_O_2_), and the hydroxyl radical (•OH) differ fundamentally in reactivity, diffusion distance, and signaling capacity [45]. O_2_^•^^−^ is membrane‐impermeable, short‐lived, and largely confined to its site of generation—the mitochondrial matrix (Complexes I and III) or the cytosolic face of the plasma membrane (NOX enzymes). By contrast, H_2_O_2_ is relatively stable, diffuses over longer distances, and crosses biological membranes through aquaporin channels, particularly AQP3 and AQP8 [46], enabling it to function as a bona fide second messenger capable of transmitting redox signals between compartments [47]. The hydroxyl radical, generated via Fenton chemistry in the presence of labile iron, reacts indiscriminately with the nearest biomolecule within nanometers of its origin and therefore cannot participate in selective signaling [45].

This compartmentalization has direct consequences for breast cancer biology. Mitochondrial ROS, once viewed solely as toxic byproducts of electron transport chain (ETC) leakage, are now recognized as regulated signaling outputs that communicate metabolic status to the cytosol and nucleus through retrograde redox signaling [48]. Mitochondrial H_2_O_2_ released to the cytosol stabilizes hypoxia‐inducible factor 1‐alpha (HIF‐1α) under hypoxia, activates nuclear factor‐κB (NF‐κB) through IκB kinase (IKK) oxidation, and modulates mitogen‐activated protein kinase (MAPK) signaling (all pathways discussed in detail in Section 4). NOX‐derived ROS at the plasma membrane, by contrast, generate spatially restricted H_2_O_2_ microdomains that selectively oxidize nearby signaling targets such as phosphatase and tensin homolog (PTEN) (Section 4.1) and receptor tyrosine phosphatases without affecting distant redox‐sensitive proteins. The resulting signaling specificity explains how the same molecule, H_2_O_2_, can simultaneously promote proliferation through membrane‐proximal phosphoinositide 3‐kinase/protein kinase B (PI3K/AKT) activation and trigger apoptosis through mitochondrial cytochrome c release, depending on where and how much is produced. Disruption of this spatial organization, through loss of compartment‐specific antioxidant defenses or altered mitochondrial dynamics (fission/fusion balance), contributes to the global redox dysregulation characteristic of breast cancer cells [49] and has implications for the design of ROS‐targeted therapies that must account for subcellular drug localization and ROS source specificity.

## 4 The Association Between Oxidative Stress and Cancer

Building on the biochemical sources and compartmentalization of ROS described in Sections 2 and 3, the following sections examine how these species are transduced into oncogenic signals through three major redox‐sensitive signaling axes.

Carcinogenesis is a multistep, multifactorial process comprising initiation, promotion, and progression [17]. ROS promote carcinogenesis [50], including in breast cancer, with reduced antioxidant levels and elevated oxidative stress detectable even before clinical symptoms of tumors emerge [12, 51]. Initiation involves irreversible DNA changes that are crucial for tumor progression [17]. Chemical carcinogens and ionizing radiation are among the most common causes of genomic instability via ROS‐mediated DNA damage [28, 52]. ROS also influence cellular behavior by interacting with membrane‐bound and intracellular receptors, modulating key signaling pathways that regulate proliferation, apoptosis, and angiogenesis [25, 50]. ROS can damage DNA in several ways. Ionizing radiation can cause double‐strand breaks both by directly damaging the DNA sugar backbone and by generating free radicals in cells, mainly hydroxyl radicals (•OH) from water [38]. ROS can also damage DNA by oxidizing bases, for example, forming 8‐oxo‐guanine, which may cause G‐to‐T transversion mutations if unrepaired [53]. Mitochondrial DNA lesions, including strand breaks and degradation, can also occur [52].

Beyond direct genomic damage, ROS also disrupt cellular function at the protein level. ROS can affect protein conformation, leading to changes in protein structure and function. The mechanism refers to oxidation of the cysteine (Cys) residues on proteins by ROS, which forms reactive sulfenic acid (–SOH) that can form disulfide bonds with nearby cysteines (–S–S–) or undergo further oxidation to sulfinic (–SO_2_H) or sulfonic acid (–SO_3_H) [54].

ROS also contribute to epigenetic dysregulation in cancer. Oxidative stress can alter DNA methylation patterns by interfering with the activity of DNA methyltransferases and by oxidizing 5‐methylcytosine to 5‐hydroxymethylcytosine via ten‐eleven translocation (TET) enzymes [55]. Furthermore, ROS modify histone proteins through changes in acetylation and methylation, thereby affecting chromatin remodeling and the expression of genes relevant to tumor initiation and progression [56].

### 4.1 Regulation of PI3K Signaling Pathways by ROS

The phosphoinositide 3‐kinase (PI3K) pathway plays an important role in cell proliferation and survival [57]. It is activated when growth factors such as epidermal growth factor (EGF), platelet‐derived growth factor (PDGF), nerve growth factor (NGF), insulin, and vascular endothelial growth factor (VEGF), cytokines, or hormones, bind to receptor tyrosine kinases (RTKs). PI3K activation leads to the phosphorylation of phosphatidylinositol‐ (4,5) bisphosphate (PIP_2_) to phosphatidylinositol (3,4,5) ‐trisphosphate (PIP_3_). The accumulation of PIP_3_ recruits AKT serine/threonine kinase (AKT), also known as protein kinase B (PKB), to the plasma membrane for phosphorylation by mechanistic target of rapamycin complex 2 (mTORC2) at Ser473, and phosphoinositide‐dependent kinase 1 (PDK1) at Thr308. PTEN dephosphorylates PIP_3_ to PIP_2_, negatively regulating the PI3K/AKT pathway [57]. Excessive oxidative stress activates the PI3K/AKT pathway by inhibiting PTEN, a tumor suppressor gene frequently altered in cancer [58, 59]. PTEN's cysteine‐rich phosphatase domain is oxidized by ROS, reducing phosphatase activity, or phosphorylated indirectly, enhancing stability and preventing membrane recruitment, resulting in constant activation of the signaling pathway [59, 60].

The regulatory mechanism involves the oxidation of cysteine residues in PTEN by H_2_O_2_ at Cys124 and Cys71 of the catalytic domain, forming an intramolecular disulfide bond that inactivates its lipid phosphatase activity [59]. PTEN oxidation is reversed by peroxiredoxin II, a cytoplasmic peroxiredoxin isoform that eliminates H_2_O_2_ generated in response to growth factors [61]. ROS also promote the translocation of NF‐κB and HIF‐1α to the nucleus, upregulating genes that support cancer growth, proliferation, and survival [17, 62].

### 4.2 NOX in Cancer and Regulation of PI3K Signaling

NOX enzymes, located in the plasma membrane or within organelle membranes, are the primary dedicated ROS‐generating enzymes in non‐phagocytic cells [29, 30]. They catalyze the reduction of molecular oxygen to superoxide anion using nicotinamide adenine dinucleotide phosphate (reduced) (NADPH) as an electron donor, yielding H_2_O_2_ and downstream species such as •OH and HOCl [30, 63]. The resulting ROS oxidize thiol groups on target proteins, modulating their function and activation status. The seven NOX family members differ in tissue distribution, subcellular localization, and regulatory subunit requirements [30]. Importantly, unlike mitochondrial ROS, which are byproducts of oxidative phosphorylation, NOX‐derived ROS are produced as the primary enzymatic function of these enzymes, allowing for spatially and temporally regulated redox signaling at specific cellular membranes and microdomains.

In breast cancer, multiple NOX isoforms are differentially expressed across molecular subtypes. NOX1, NOX2, and NOX4 are generally overexpressed in breast tumors compared to normal tissue, although NOX4 expression is notably lower in the basal subtype. NOX2, NOX4, and NOX5 mRNA levels increase with tumor progression stage, and low expression of NOX1, NOX4, and DUOX1 is associated with unfavorable prognosis [64]. NOX1 has been specifically implicated in estrogen receptor‐positive breast cancer, where estrogen‐induced NOX1 upregulation promotes oxidative stress and downregulation of the anti‐apoptotic protein survivin, contributing to tumor initiation [65]. NOX4 is overexpressed in several malignancies beyond breast cancer, including kidney and non‐small cell lung cancer, promoting proliferation and metastasis [29, 63].

**Figure 3:**
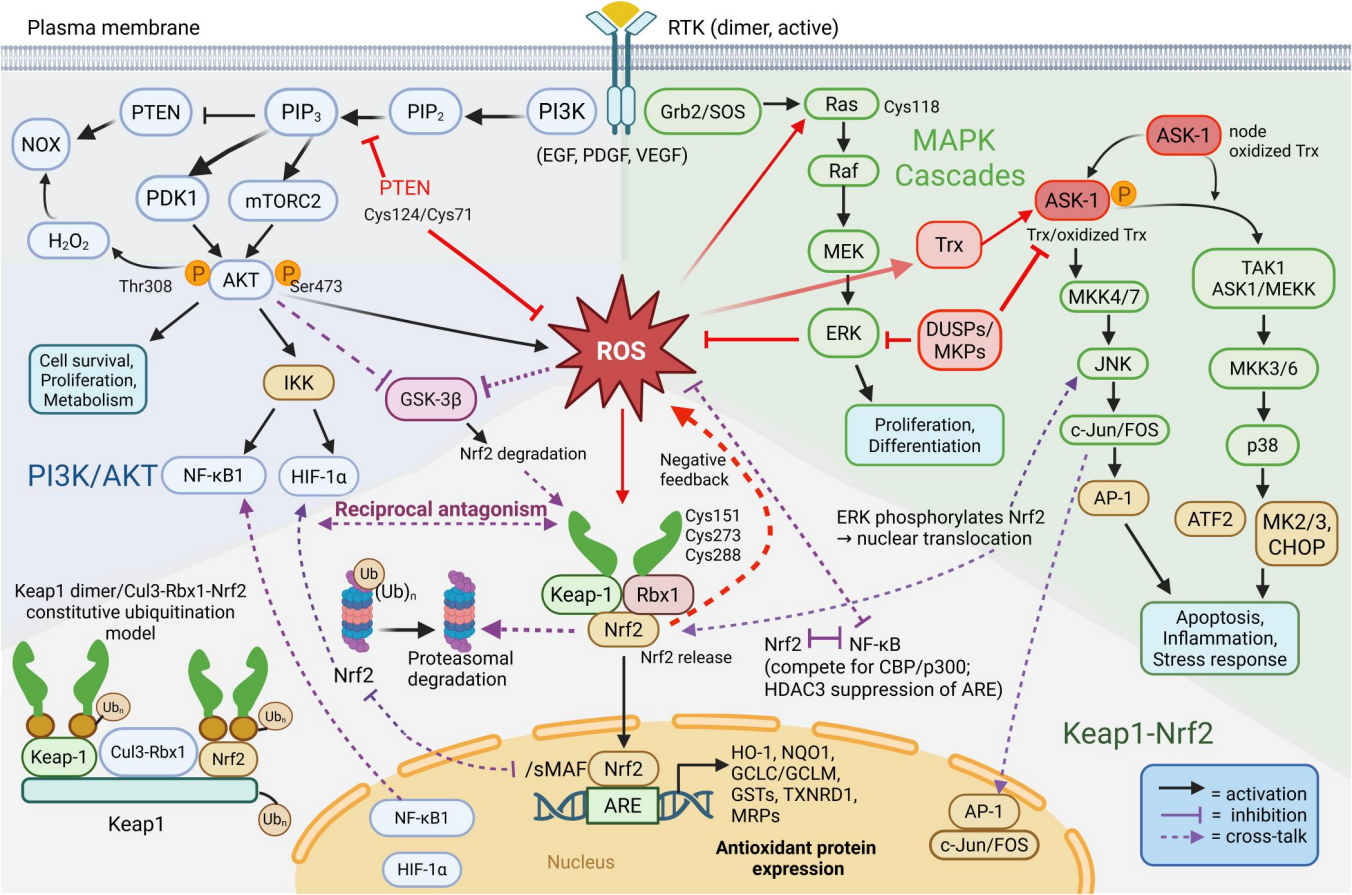
ROS‐mediated signaling pathway cross‐talk in breast cancer. The three major ROS‐regulated signaling axes—PI3K/AKT (left), MAPK cascades (right), and Keap1‐Nrf2 (bottom)—are shown within a shared cellular context to illustrate their functional interconnections. ROS (center) acts as a master upstream regulator by oxidatively inactivating PTEN (sustaining PI3K/AKT signaling), activating Ras via Cys118 oxidation, inactivating DUSPs to prolong ERK signaling, releasing ASK1 from thioredoxin to activate JNK/p38 stress kinases, and modifying Keap1 sensor cysteines to stabilize Nrf2. Cross‐talk connections (purple dashed arrows) highlight: AKT‐mediated inhibition of GSK‐3β, which prevents Nrf2 phosphorylation and degradation; ERK‐mediated phosphorylation promoting Nrf2 nuclear translocation; reciprocal antagonism between Nrf2 and NF‐κB through competition for the transcriptional coactivator CBP/p300 and HDAC3‐mediated suppression of ARE‐driven transcription; and the negative feedback loop whereby Nrf2‐driven antioxidant gene expression reduces intracellular ROS levels. All depicted transcription factors (Nrf2/sMAF, AP‐1, NF‐κB, HIF‐1α) converge on a shared nucleus, reflecting the integrated nature of redox‐regulated transcriptional reprogramming in breast cancer cells. ARE, antioxidant response element; ASK1, apoptosis signal‐regulating kinase 1; DUSPs, dual‐specificity phosphatases; GSK‐3β, glycogen synthase kinase 3 beta; HIF‐1α, hypoxia‐inducible factor 1 alpha; MAPK, mitogen‐activated protein kinase; MKPs, MAPK phosphatases; NF‐κB, nuclear factor kappa B; NOX, NADPH oxidase; Nrf2, nuclear factor erythroid 2‐related factor 2; PI3K, phosphoinositide 3‐kinase; PTEN, phosphatase and tensin homolog; ROS, reactive oxygen species; sMAF, small musculoaponeurotic fibrosarcoma protein; Trx, thioredoxin. Created in BioRender. M. Nikolovska, 2026, https://BioRender.com/0ptx33x.

The connection between NOX enzymes and the PI3K pathway constitutes a self‐amplifying oncogenic circuit. PI3K activation converts PIP_2_ to PIP_3_, which stimulates NOX‐derived H_2_O_2_ production. This H_2_O_2_ in turn oxidizes and inactivates PTEN's lipid phosphatase activity, preventing the dephosphorylation of PIP_3_ back to PIP_2_ and thereby sustaining PI3K/AKT signaling [59, 62]. Additionally, AKT can positively regulate NOX4 expression through NF‐κB‐mediated transcriptional activation, establishing a positive feedback loop that drives constitutive pathway activation [66]. This NOX–PI3K feedforward mechanism is illustrated in Figure 3.

### 4.3 MAPK Cascades

The MAPK family includes extracellular signal‑regulated kinases (ERK), c‐Jun N‐terminal kinases (JNKs), and p38 mitogen‐activated protein kinases (p38s), and three to five kinase tiers (MAP4K, MAP3K, MAPKK, MAPK, and MAPKAPK) [67]. The ERK pathway (Figure 3) is activated by stimuli such as EGF, tumor necrosis factor (TNF), activators of protein kinase C (PKC), and Src family members, which bind to the receptor. Growth factor receptor‑binding protein 2 (Grb2) binds to the activated receptors, recruiting the receptor‑Grb2‑SOS complex (Son of Sevenless‐SOS). This leads to translocation of the cytoplasmic SOS to the membrane, resulting in a high concentration of SOS near Ras. SOS exchanges GDP for GTP on Ras, activating Raf‑1 protein kinase (MAP3K), which phosphorylates downstream MEK (MAPKK), then ERKs (MAPK). Dimerized ERK translocates to the nucleus, where it regulates transcription factors such as proto‑oncogene c‑Fos, proto‑oncogene c‑Jun, ETS domain‑containing protein Elk‑1, proto‑oncogene c‑Myc, and cyclic AMP‑dependent transcription factor ATF2 [67]. Elevated ERK expression has been detected in various human tumors as well as breast cancer [67, 68].

ROS can activate the ERK pathway at multiple levels [69]. At the receptor level, ROS oxidize cysteine‐rich motifs on receptor tyrosine kinases, enabling ligand‐independent activation. ROS can also directly oxidize Ras at Cys118, promoting its GTP‐bound active state and thereby initiating the Raf–MEK–ERK cascade independently of growth factor signaling [70]. Additionally, ROS inactivate MAPK phosphatases (MKPs/DUSPs), the cysteine‐dependent phosphatases that normally dephosphorylate and deactivate ERK, thus prolonging MAPK signaling [69, 71]. Consistent with this bidirectional relationship, Acosta‐Casique et al. demonstrated that treatment with PD0325901, a MEK inhibitor, decreased both ROS levels and mitochondrial fragmentation in MDA‐MB‐231 TNBC cells, indicating that active MAPK signaling itself sustains elevated ROS in breast cancer cells [72].

JNK and p38 pathways (Figure 3) respond to environmental and genotoxic stressors, cytokines, inflammatory factors, and growth factors [67, 73]. MAP3Ks (e.g., ASK1, HPK1, and MLK‐3) are upstream kinases activated by cellular stress that phosphorylate MAPKKs (MKK4 and MKK7), which then activate JNK [74, 75]. JNK phosphorylation further activates c‐Jun, Activator Protein‐1 (AP‐1), and FBJ murine osteosarcoma viral oncogene homolog (FOS) [69, 74]. In p38 activation, upstream MAP3Ks (TAK1, ASK1, and MEKK1–4) activate MAPKKs (MKK3 and MKK6), which then phosphorylate p38 and its downstream substrates (ATF2, CHOP, MK2, and MK3) [73]. The JNK/p38 MAPK pathways often act as tumor suppressors but can also function as tumor promoters under certain conditions, depending on the cellular context and the duration of pathway activation [73, 75, 76].

### 4.4 Keap1–Nrf2 Antioxidative Pathway

Nuclear factor‐erythroid‐2‐related factor 2 (Nrf2) is the master transcriptional regulator of the cellular antioxidant and cytoprotective response. Under basal conditions, Nrf2 protein levels are kept low through continuous ubiquitination and proteasomal degradation mediated by Kelch‐like ECH‐associated protein 1 (Keap1), which functions as a substrate adapter for the Cullin 3 (Cul3)–Rbx1 E3 ubiquitin ligase complex [77, 78]. Keap1 contains three functional domains: the BTB (broad complex/tramtrack/bric‐a‐brac) domain, which mediates Keap1 homodimerization and Cul3 recruitment, thereby enabling Nrf2 ubiquitination and degradation; the intervening region (IVR), which harbors reactive cysteine residues (particularly Cys151, Cys273, and Cys288) that serve as redox sensors detecting electrophiles and oxidants; and the Kelch/DGR (double glycine repeat) domain, which directly binds the ETGE and DLG motifs in the Neh2 domain of Nrf2 sequestering it for proteasomal turnover [77, 79]. Keap1 possesses over 25 cysteine residues, of which three—Cys151 in the BTB domain and Cys273 and Cys288 in the IVR—serve as the principal electrophile and oxidant sensors [80]. Modification of these residues by electrophiles or ROS induces conformational changes in Keap1 that impair its ubiquitin ligase activity, without necessarily causing full Keap1–Nrf2 dissociation. As a result, newly synthesized Nrf2 escapes degradation, accumulates, and translocates to the nucleus, where it heterodimerizes with small Maf proteins and binds to antioxidant response elements (AREs) in the promoters of cytoprotective genes (Figure 3) [77, 78]. Importantly, different electrophiles target distinct cysteine residues (a “cysteine code”), enabling Keap1 to discriminate among diverse stress stimuli and mount tailored responses [80].

Nrf2 target genes encompass a broad cytoprotective program extending well beyond classical antioxidant enzymes. These include heme oxygenase‐1 (HO‐1), NAD(P)H: quinone oxidoreductase 1 (NQO1), glutathione S‐transferases (GSTs), superoxide dismutase (SOD), catalase (CAT), and glutathione reductase, as well as enzymes critical for glutathione biosynthesis (glutamate‐cysteine ligase catalytic (GCLC) and modifier (GCLM) subunits) and the thioredoxin/thioredoxin reductase (TXNRD1) system. In the context of cancer, Nrf2 also upregulates multidrug resistance‐associated proteins (MRPs/ABCCs), which contribute to drug efflux and chemoresistance, and metabolic enzymes in the pentose phosphate pathway and serine biosynthesis that support anabolic demands of proliferating tumor cells [77, 81].

A critical feature of Nrf2 signaling in the context of cancer is its paradoxical dual role. In normal cells and during early carcinogenesis, transient Nrf2 activation is unequivocally cytoprotective: it detoxifies carcinogens, neutralizes ROS, and suppresses tumor initiation [81, 82]. However, once tumors are established, constitutive Nrf2 activation confers a selective advantage to cancer cells by enhancing their antioxidant capacity, promoting metabolic reprogramming, and enabling resistance to chemotherapy and radiotherapy—a phenomenon described as “Nrf2 addiction” [81]. In cancer, constitutive Nrf2 activation arises through multiple mechanisms: somatic mutations in *KEAP1* (loss‐of‐function), *NFE2L2* (gain‐of‐function in the ETGE/DLG motifs), or *CUL3*; epigenetic silencing of *KEAP1* by promoter hypermethylation; oncogene‐driven transcriptional upregulation of *NFE2L2* by *KRAS*, *BRAF*, and *MYC*; stabilization of Nrf2 through PI3K/AKT‐mediated inhibition of GSK‐3β/β‐TrCP‐dependent degradation; and accumulation of p62/SQSTM1 following autophagy disruption, which sequesters Keap1 and liberates Nrf2 [78, 82]. In breast cancer specifically, constitutive Nrf2 activation has been associated with poor prognosis and enhanced chemoresistance. Nrf2 overexpression confers resistance to doxorubicin and cisplatin through upregulation of antioxidant enzymes and drug efflux transporters [82, 83], while Nrf2‐mediated induction of thioredoxin, peroxiredoxin, and glutamate‐cysteine ligase has been implicated in tamoxifen resistance in ER‐positive breast cancer cells [82]. A pan‐cancer machine learning analysis of Nrf2 pathway‐related genes has further confirmed the prognostic significance of this pathway across tumor types and identified novel Nrf2‐associated gene networks with potential therapeutic implications [84].

The cytoprotective function of the Keap1‐Nrf2 pathway intersects extensively with the NF‐κB inflammatory signaling pathway. Under oxidative stress, IκB kinases (IKK) are activated, leading to phosphorylation and degradation of IκBα, release of NF‐κB (p65/p50) heterodimers, and their nuclear translocation to drive transcription of pro‐inflammatory mediators including interleukin‐6 (IL‐6), interleukin‐1β (IL‐1β), tumor necrosis factor α (TNF‐α), and inducible nitric oxide synthase (iNOS) [85]. Nrf2 opposes NF‐κB‐driven inflammation through multiple mechanisms: by inducing HO‐1, which exerts anti‐inflammatory effects; by upregulating antioxidant enzymes that reduce the ROS required for sustained IKK activation; and by competing with NF‐κB p65 for the transcriptional coactivator CBP/p300, thereby directly restraining NF‐κB transcriptional activity [86, 87]. Conversely, NF‐κB p65 can suppress Nrf2 target gene expression by recruiting histone deacetylase 3 (HDAC3) to ARE sequences, establishing a reciprocal antagonism between these two pathways that has important implications for the inflammatory tumor microenvironment [86].

Additionally, the DNA damage response (DDR) interfaces with Nrf2 signaling through the tumor suppressor BRCA1. BRCA1 physically interacts with the ETGE motif of Nrf2, competing with Keap1 for Nrf2 binding, thereby inhibiting Keap1‐mediated Nrf2 ubiquitination and promoting Nrf2 activation and downstream antioxidant gene expression [12]. This BRCA1–Nrf2 axis has particular relevance to breast cancer: loss of BRCA1 function reduces Nrf2‐dependent antioxidant capacity, leading to increased oxidative stress and genomic instability, while paradoxically rendering BRCA1‐deficient cells more dependent on alternative ROS‐scavenging mechanisms. Poly(ADP‐ribose) polymerase 1 (PARP1), which operates in a separate DNA repair pathway (single‐strand break repair) from BRCA1 (homologous recombination), has also been reported to act as an Nrf2 transcriptional coactivator through binding to the small Maf protein MafG, enhancing Nrf2‐driven gene expression [82, 85].

### 4.5 ROS‐Mediated DNA Damage and Mutagenesis

As outlined above, ROS induce diverse forms of DNA damage; this section discusses these mechanisms and their downstream consequences in greater detail. ROS‐mediated DNA damage includes base modifications, sugar lesions, single‐ and double‐strand breaks, abasic sites, and DNA–protein crosslinks, many of which possess mutagenic potential [88, 89]. Guanine is particularly susceptible to oxidation owing to its low redox potential, yielding 8‐oxo‐7,8‐dihydroguanine (8‐oxo‐dG), the most abundant and best‐characterized oxidative DNA lesion. During replication, 8‐oxo‐dG mispairs with adenine, predominantly causing G‐to‐T transversion mutations (corresponding to C‐to‐A on the complementary strand), which are among the most frequent somatic mutations observed across human cancers [89, 90]. Other oxidative lesions, including 8‐oxo‐dA, 5‐hydroxycytosine, and thymine glycol, are similarly mutagenic [88]. At the genomic level, high ROS concentrations can activate oncogenes (e.g., *KRAS*) and inactivate tumor suppressors (e.g., *TP53*) through oxidative mutagenesis, thereby initiating and promoting carcinogenesis [90].

The DNA damage repair (DDR) system counters these effects through multiple pathways: base excision repair (BER) for oxidized bases and abasic sites, nucleotide excision repair (NER) for bulky adducts and intra‐strand crosslinks, mismatch repair (MMR) for replication errors including those caused by oxidized template bases, and homologous recombination (HR) or non‐homologous end joining (NHEJ) for double‐strand breaks [89, 91]. Importantly, ROS not only generate DNA lesions but also compromise the repair machinery itself. ROS promote the proteasomal degradation of BRCA2, a key mediator of HR, thereby impairing high‐fidelity double‐strand break repair and channeling repair toward the error‐prone NHEJ pathway [92]. Additionally, oxidative inactivation of the mismatch repair protein MSH6 and the BER glycosylase OGG1 has been reported under conditions of sustained oxidative stress, creating a self‐reinforcing loop of accumulating unrepaired damage [90]. Thus, ROS simultaneously generate DNA lesions and impair the cellular capacity to repair them, accelerating mutation accumulation and tumor evolution.

### 4.6 ROS‐Driven Post‐Translational Modifications (PTMs) and Cell Fate Determination

Beyond genomic damage, ROS exert pervasive effects on cellular function through direct PTM of proteins, altering their structure, activity, localization, and interactions [93]. The principal ROS‐sensitive residue is cysteine, whose thiol side chain (–SH) can undergo a hierarchy of oxidative modifications with increasing severity: reversible sulfenylation (–SOH), S‐glutathionylation (–SSG), and disulfide bond formation (–S–S–), as well as the largely irreversible sulfinylation (–SO_2_H) and sulfonylation (–SO_3_H) [54]. This redox sensitivity of cysteine residues underpins the signaling functions of many of the pathways discussed above. For example, ROS‐mediated oxidation of catalytic cysteines in protein tyrosine phosphatases (including PTEN, as discussed in Section 4.2) and MAPK phosphatases (DUSPs, Section 4.3) transiently inactivates these negative regulators, amplifying kinase‐driven oncogenic signaling. S‐glutathionylation, the reversible conjugation of glutathione to protein cysteines, represents a major protective mechanism against irreversible oxidation while also serving as a redox‐sensitive signaling switch for proteins including Ras, NF‐κB p65, and caspase‐3 [94, 95].

Protein carbonylation, the irreversible introduction of carbonyl groups (aldehydes and ketones) into amino acid side chains (particularly Pro, Arg, Lys, and Thr), represents a hallmark of severe oxidative damage. Unlike reversible thiol modifications that participate in signaling, carbonylation typically marks proteins for proteasomal degradation and is widely used as a biomarker of oxidative stress in cancer tissues, including breast tumors [93, 96]. The tumor suppressor p53, a central integrator of stress responses, is subject to extensive redox‐dependent PTMs. ROS facilitate p53 stabilization and activation through phosphorylation by redox‐sensitive kinases including ATM, p38α MAPK, and ERK, which respond to oxidative DNA damage. Acetylation, methylation, and ubiquitylation further fine‐tune p53 transcriptional activity and protein stability in a context‐dependent manner [97]. Beyond individual proteins, ROS‐mediated lipid peroxidation generates reactive aldehydes such as malondialdehyde (MDA) and 4‐hydroxynonenal (4‐HNE), which form covalent adducts with proteins and DNA, amplifying oxidative damage and contributing to mutagenesis and disruption of membrane integrity [13, 98]. In breast cancer, elevated levels of lipid peroxidation markers have been detected in patient serum and tumor tissue, supporting their role as both contributors to carcinogenesis and potential biomarkers [98]. Furthermore, recent machine learning approaches using panels of oxidative stress biomarkers, including MDA, total antioxidant capacity, SOD, CAT, and GPx, have demonstrated high accuracy in classifying breast cancer status, supporting the diagnostic potential of redox profiling [99]. Lipid peroxidation is also the central executory mechanism of ferroptosis, an iron‐dependent form of regulated cell death driven by the accumulation of lipid peroxides, linking this pathway directly to the redox dysregulation observed in breast cancer [100].

The cumulative impact of ROS on cellular fate is ultimately determined by their concentration, duration, and subcellular localization. At physiological levels, ROS function as essential signaling molecules that support proliferation, differentiation, and adaptive stress responses. Moderate sustained elevations, characteristic of the tumor microenvironment, promote malignant transformation through oncogenic signaling, mutagenesis, and PTM‐mediated pathway dysregulation described above. However, when ROS accumulation exceeds the capacity of cellular antioxidant defenses, the balance tips toward cell death. Excessive ROS trigger apoptosis through multiple convergent mechanisms: direct oxidation of mitochondrial membrane lipids promotes mitochondrial outer membrane permeabilization and cytochrome c release; ROS shift the balance of Bcl‐2 family proteins toward pro‐apoptotic members (Bax/Bak); and oxidative activation of ASK1 (via thioredoxin dissociation, as discussed in Section 4.3) drives sustained JNK and p38 activation that promotes apoptotic gene expression [101]. Cancer cells adapt to their elevated baseline ROS by upregulating antioxidant systems, including the Nrf2‐driven program discussed in Section 4.4, but this adaptation creates a therapeutic vulnerability: pro‐oxidant strategies that further increase ROS or inhibit antioxidant defenses can selectively push cancer cells past the apoptotic threshold while sparing normal cells that operate at lower baseline ROS levels (Figure 1) [12, 90]. This principle underlies many of the therapeutic approaches discussed in subsequent sections.

## 5 Antioxidant‐Based Therapeutic Strategies

**Figure 4:**
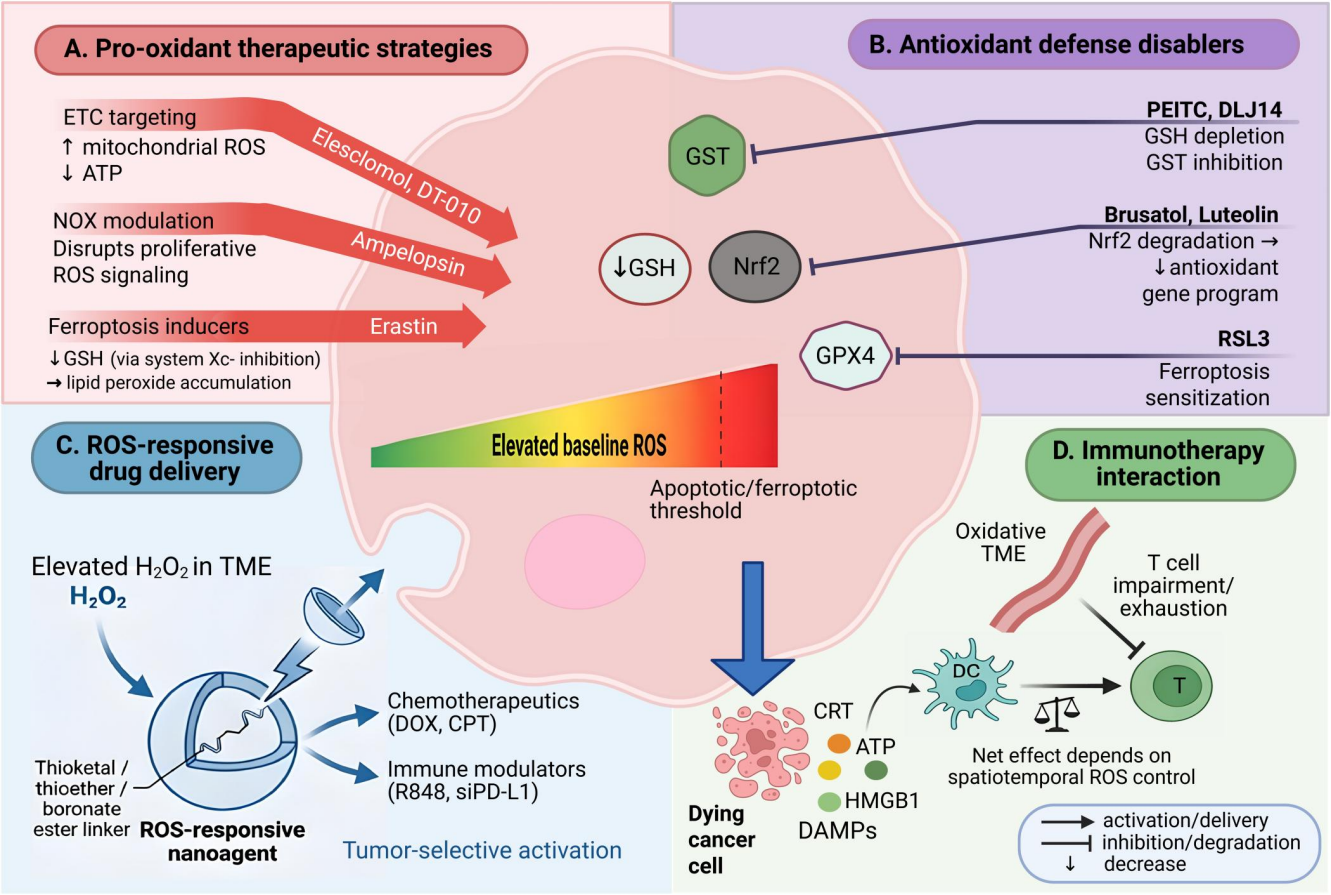
Therapeutic strategies exploiting redox vulnerabilities in breast cancer. Cancer cells maintain elevated baseline ROS through upregulated antioxidant defenses (Nrf2, GSH, and GPX4), creating a narrow therapeutic window. (A) Pro‐oxidant strategies (ETC inhibitors and ferroptosis inducers) and (B) antioxidant defense disablers (GSH depleters and Nrf2 inhibitors) converge to push cancer cells past the apoptotic/ferroptotic threshold. (C) ROS‐responsive nanoagents achieve tumor‐selective drug release through cleavage of ROS‐sensitive linkers (thioketals, thioethers, and boronate esters) in the oxidative tumor microenvironment. (D) At the immunotherapy interface, ROS‐driven cell death releases DAMPs (calreticulin, ATP, and HMGB1) that prime antitumor immunity, while oxidative stress in the TME simultaneously impairs effector T‐cell function—a paradox requiring spatiotemporal ROS control for optimal therapeutic outcomes. CRT, calreticulin; DAMPs, damage‐associated molecular patterns; DC, dendritic cell; DOX, doxorubicin; CPT, camptothecin; ETC, electron transport chain; GPX4, glutathione peroxidase 4; GSH, glutathione; GST, glutathione S‐transferase; HMGB1, high mobility group box 1; Nrf2, nuclear factor erythroid 2‐related factor 2; PEITC, phenethyl isothiocyanate; ROS, reactive oxygen species; TME, tumor microenvironment. Created in BioRender. R. Stojchevski, 2026, https://BioRender.com/4lb9chi.

The signaling pathways described in Section 4 represent potential therapeutic targets, but an equally important clinical question is whether modulating systemic antioxidant status can influence breast cancer outcomes. The preceding sections established that the biological outcome of ROS signaling is concentration‐dependent: moderate elevations promote tumor progression, while excessive accumulation triggers cancer cell death. This duality creates both a rationale and a dilemma for antioxidant‐based therapeutic strategies. On one hand, antioxidants may suppress cancer initiation by reducing oxidative DNA damage and mutagenesis; on the other, they may inadvertently protect established tumors by reinforcing cancer cell antioxidant defenses and diminishing the efficacy of ROS‐generating therapies such as radiotherapy and certain chemotherapeutics [102, 103]. Large‐scale chemoprevention trials, including SELECT (vitamin E and selenium in prostate cancer) and the ATBC trial (β‐carotene in lung cancer among smokers), underscored this complexity by demonstrating that antioxidant supplementation can, under certain conditions, increase rather than decrease cancer incidence [103, 104] (Figure 4).

This section evaluates the mechanisms, preclinical evidence, and translational challenges of dietary and endogenous antioxidants with reported activity in breast cancer models, while recognizing that the therapeutic window for antioxidant intervention remains incompletely defined. It must be emphasized that the evidence base for antioxidant interventions in breast cancer is dominated by preclinical cell culture and animal studies, with very few adequately powered randomized controlled trials in breast cancer patients. The risk‐benefit calculus differs fundamentally between chemoprevention in healthy individuals, adjuvant use during active treatment, and supportive care in survivorship, and conclusions drawn from one context should not be extrapolated to another.

### 5.1 Melatonin

Melatonin (N‐acetyl‐5‐methoxytryptamine), an indoleamine hormone synthesized primarily by the pineal gland, functions as both a direct free radical scavenger and an indirect antioxidant with pleiotropic anticancer properties. Melatonin and its metabolites (N1‐acetyl‐N2‐formyl‐5‐methoxykynuramine [AFMK] and N1‐acetyl‐5‐methoxykynuramine [AMK]) directly neutralize •OH, O_2_^−^•, and ONOO^−^ through electron donation, and melatonin additionally upregulates antioxidant enzymes including SOD, GPx, and glutathione reductase while activating Nrf2‐dependent cytoprotective gene expression [105, 106]. Notably, melatonin accumulates in mitochondria at concentrations far exceeding plasma levels, where it reduces electron leakage from Complexes I and III, protects mitochondrial membrane potential, and limits mitochondrial ROS production [107]. These antioxidant mechanisms underpin its antiproliferative, antimetastatic, and proapoptotic effects observed in breast cancer models [108].

Melatonin exerts receptor‐mediated effects through the G‐protein‐coupled receptors MT1 and MT2 [109]. Activation of MT1 inhibits adenylate cyclase, reducing intracellular cAMP levels and consequently diminishing protein kinase A (PKA)‐mediated phosphorylation of estrogen receptor α (ERα). This attenuation of ERα transcriptional activity, including reduced binding to estrogen‐response elements in target genes such as cyclin D1, offers a mechanistic basis for melatonin's selective efficacy against ER‐positive breast cancers, paralleling the action of anti‐estrogen agents like tamoxifen [110, 111]. Melatonin also destabilizes HIF‐1α, thereby suppressing VEGF‐driven angiogenesis and reducing the hypoxia‐adapted phenotype that sustains tumor progression [109]. Melatonin secretion follows a circadian rhythm, peaking nocturnally; disruption of this rhythm through night‐shift work or chronic light‐at‐night exposure suppresses melatonin secretion and has been epidemiologically associated with increased breast cancer risk, although the magnitude of this association remains debated in large prospective cohorts [110, 112].

Despite promising preclinical data, including evidence from rodent xenograft models demonstrating that physiological nocturnal melatonin levels reduce tumor proliferation, linoleic acid uptake, and MEK/ERK signaling [110], clinical translation remains limited. Most human studies are small, of short duration, or lack randomized controlled designs, and the protective efficacy of melatonin supplementation in cancer patients or high‐risk individuals has not been established in large‐scale trials [108]. Key aspects of melatonin in breast cancer are summarized in Table 1.

**Table 1:** Comparative summary of antioxidants evaluated in breast cancer: Redox mechanisms, preclinical and clinical evidence, translational limitations, and key references.

Antioxidant	Key redox mechanisms	Breast cancer evidence	Clinical limitations	Key refs.
Melatonin	Direct •OH/O_2_^−^•/ONOO^−^ scavenger; Nrf2 activation; mitochondrial accumulation (↓Complex I/III leakage); MT1/MT2 → ↓ cAMP → attenuated ERα activity; HIF‐1α destabilization	Antiproliferative/proapoptotic in ER+ models; circadian disruption linked to ↑ breast cancer risk (IARC 2A)	No large‐scale RCTs; night‐shift risk magnitude debated	[105, 106, 107, 108, 110, 111]
Curcumin	Low dose: radical scavenger, Nrf2 activator (Keap1 Cys modification). High dose: pro‐oxidant (Michael acceptor, GSH depletion, TrxR inhibition); NF‐κB/IKK inhibition; ↓ PI3K/AKT/mTOR	Reduces tumor volume; synergy with paclitaxel/doxorubicin; ↓ proliferation, migration, metastasis	Poor bioavailability; β‐diketone instability; MACs and nanoformulations preclinical only; no breast cancer RCTs	[113, 114, 115, 116, 117, 118]
Vitamin C (Ascorbic acid)	Oral (μM): electron donor; regenerates α‐tocopherol; cofactor for HIF‐PHDs and TET dioxygenases; IV (mM): pro‐oxidant via Fe‐catalyzed autoxidation → extracellular H_2_O_2_	IV doses selectively cytotoxic to cancer cells; dietary intake inversely associated with breast cancer mortality in some meta‐analyses	SELECT concerns; isoform‐specific effects poorly characterized clinically; no tocotrienol breast cancer trials	[119, 120, 121, 122, 123]
Vitamin E (Tocopherols and Tocotrienols)	Chain‐breaking LOO• scavenger (recycled by ascorbate); α‐tocopherol suppresses ferroptosis (GPX4‐independent). Tocotrienols: ↓ NF‐κB, PI3K/AKT, STAT3; pro‐oxidant at high dose; ↓ MDR1/P‐gp	Tocotrienols (γ, δ) > tocopherols for growth suppression; selective toxicity versus normal cells; resensitizes drug‐resistant cells.	SELECT concerns; isoform‐specific effects poorly characterized clinically; no tocotrienol breast cancer trials	[124, 125, 126, 127, 128, 129, 130, 131, 132]
Carotenoids	^1^O_2_ quenching (energy transfer); O_2_‐tension‐dependent switch: antioxidant at normal pO_2_, pro‐oxidant under tumor hypoxia; ↓ PI3K/AKT, NF‐κB; ↑ p53, p27	β‐Carotene: S‐phase arrest in TNBC; lycopene: inverse association with breast cancer risk (meta‐analysis); lutein: enhances taxane cytotoxicity	Low solubility/bioavailability; ATBC/CARET safety concerns; breast cancer‐specific clinical data lacking	[133, 134, 135, 136, 137]

*Note:* Each compound exhibits concentration‐dependent duality, functioning as an antioxidant at low doses and as a pro‐oxidant at pharmacological concentrations. Detailed mechanistic discussions are provided in Sections 5.1, 5.2, 5.3, 5.4, 5.5 of the main text.

Abbreviations: ATBC, Alpha‐Tocopherol, Beta Carotene Cancer Prevention Study; CARET, Beta‐Carotene and Retinol Efficacy Trial; ER+, estrogen receptor‐positive; GSH, glutathione; GPX4, glutathione peroxidase 4; HIF‐PHD, hypoxia‐inducible factor prolyl hydroxylase; IARC, International Agency for Research on Cancer; IV, intravenous; LOO•, lipid peroxyl radical; MACs, monocarbonyl analogs of curcumin; MDR1, multidrug resistance protein 1; P‐gp, P‐glycoprotein; RCT, randomized controlled trial; SELECT, Selenium and Vitamin E Cancer Prevention Trial; TET, ten‐eleven translocation dioxygenase; TNBC, triple‐negative breast cancer; TrxR, thioredoxin reductase.

### 5.2 Curcumin

Curcumin (diferuloylmethane), the principal polyphenol of *Curcuma longa*, exhibits concentration‐dependent redox activity that is directly relevant to the ROS‐mediated mechanisms discussed above. At low concentrations, curcumin functions as a direct free radical scavenger through hydrogen atom donation from its phenolic groups, and as an indirect antioxidant by activating Nrf2: curcumin modifies Keap1 cysteine residues, promoting Nrf2 nuclear translocation and upregulation of HO‐1, NQO1, and glutathione biosynthetic enzymes [113, 138, 139]. At higher pharmacological concentrations, however, curcumin acts as a pro‐oxidant, generating ROS through redox cycling of its α,β‐unsaturated carbonyl (Michael acceptor) moiety, depleting intracellular GSH, and inhibiting thioredoxin reductase [114]. In cancer cells with already elevated baseline ROS, this additional oxidative burden can exceed the apoptotic threshold, selectively inducing cell death while sparing normal cells that operate at lower ROS levels [113, 140]. This pro‐oxidant mechanism converges with curcumin's well‐characterized suppression of NF‐κB signaling; that is, curcumin inhibits IKK activity and blocks IκBα phosphorylation, reducing NF‐κB‐driven expression of anti‐apoptotic (Bcl‐2, Bcl‐xL, and survivin) and pro‐inflammatory (COX‐2, IL‐6, and TNF‐α) genes [115, 116]. Additional downstream effects include downregulation of PI3K/AKT/mTOR signaling, stabilization and activation of wild‐type p53 with upregulation of Bax and p21, and inhibition of STAT3 and cyclin D1, collectively suppressing proliferation and inducing apoptosis in breast cancer models [117, 140].

Despite this broad‐spectrum anticancer activity in preclinical systems, curcumin's clinical translation is severely limited by poor oral bioavailability, rapid hepatic and intestinal metabolism, chemical instability of the β‐diketone moiety under physiological conditions, and low aqueous solubility [118, 141]. To address these pharmacokinetic limitations, two complementary strategies have been pursued. First, structural modification: monocarbonyl analogs of curcumin (MACs) replace the labile β‐diketone with a stable single carbonyl group, improving metabolic stability while retaining or enhancing anticancer potency through NF‐κB and MAPK pathway inhibition [142, 143, 144]. Recent work from our group has demonstrated that curcumin and its monocarbonyl analogs C66 and B2BrBC modulate oxidative stress biomarkers in a cell type‐specific manner, with opposing effects on SOD and CAT activity in MDA‐MB‐231 cancer cells versus non‐tumorigenic MCF‐10A cells [145]. Second, advanced drug delivery systems, such as nano‐micelles, solid lipid nanoparticles, polymeric nanoparticles, and folic acid‐modified liposomes, aim to improve tumor‐targeted delivery, enhance bioavailability, and enable combination regimens with standard chemotherapeutics (e.g., paclitaxel and doxorubicin) [146]. Curcumin‐based combination therapies have shown synergistic effects in preclinical breast cancer models, particularly in overcoming drug resistance, although translation to clinical application remains limited by the absence of large‐scale randomized controlled trials [115, 147]. A summary of curcumin's mechanisms and challenges is provided in Table 1.

### 5.3 Vitamin C

Vitamin C (ascorbic acid) is a water‐soluble antioxidant whose effects in cancer are critically dependent on concentration and route of administration. At physiological concentrations (micromolar range, achievable through oral intake), ascorbate acts as a classical antioxidant: it donates electrons to neutralize free radicals, regenerates α‐tocopherol from its tocopheroxyl radical form, and serves as an essential cofactor for Fe^2+^/2‐oxoglutarate‐dependent dioxygenases [119]. Among these dioxygenases, two families are particularly relevant to cancer. First, HIF prolyl hydroxylases (PHD1–3) require ascorbate to hydroxylate HIF‐1α, targeting it for proteasomal degradation; ascorbate deficiency in tumors may therefore contribute to HIF‐1α stabilization and hypoxia‐driven angiogenesis, metastasis, and chemoresistance [148]. Second, TET DNA dioxygenases use ascorbate as a cofactor to oxidize 5‐methylcytosine to 5‐hydroxymethylcytosine, initiating active DNA demethylation. Vitamin C‐mediated restoration of TET activity can reactivate epigenetically silenced tumor suppressor genes, representing a mechanism that connects redox biology to epigenetic reprogramming in cancer [120].

At pharmacological concentrations (millimolar range, achievable only through intravenous administration), ascorbate undergoes a functional switch from antioxidant to pro‐oxidant. At these supraphysiological levels, ascorbate autoxidizes in the extracellular space through catalysis by free transition metals (particularly Fe^3+^/Fe^2+^), generating H_2_O_2_ at concentrations sufficient to induce oxidative cytotoxicity [119, 121]. Cancer cells are selectively vulnerable to this mechanism because they harbor elevated labile iron pools and frequently exhibit reduced catalase expression relative to normal tissues, impairing their capacity to detoxify the ascorbate‐generated H_2_O_2_ [121]. This pharmacological pro‐oxidant effect, essentially using vitamin C as an H_2_O_2_ delivery prodrug, is mechanistically distinct from the dietary antioxidant role and cannot be achieved through oral supplementation, which is limited to ~200 μM plasma concentrations by saturable intestinal absorption and renal excretion [119].

Epidemiological evidence on dietary vitamin C and breast cancer risk has been inconsistent, with some studies reporting reduced breast cancer‐specific mortality associated with higher dietary intake [122], while others found no significant association [149, 150]. Importantly, these dietary studies address micromolar‐range exposures and do not inform the pharmacological IV vitamin C paradigm. Early‐phase clinical trials of high‐dose IV ascorbate in combination with standard chemotherapy have demonstrated safety and tolerability in advanced breast cancer, with preliminary evidence of improved quality of life, though definitive efficacy data from randomized trials are lacking [123]. Table 1 summarizes ascorbic acid's functions, mechanisms, and limitations in breast cancer.

### 5.4 Vitamin E

Vitamin E encompasses eight naturally occurring lipophilic chromanols classified into two groups ‐ tocopherols (α, β, γ, and δ) and tocotrienols (α, β, γ, and δ), distinguished by the saturation of their phytyl side chain [151]. The primary biochemical function of all vitamin E isoforms is chain‐breaking antioxidant activity in lipid bilayers: the chromanol hydroxyl group donates a hydrogen atom to lipid peroxyl radicals (LOO•), terminating lipid peroxidation chain reactions and generating the relatively stable tocopheroxyl radical, which is recycled by ascorbate (as discussed in Section 5.3) [124]. This lipid peroxidation‐suppressing activity is directly relevant to ferroptosis, the iron‐dependent cell death pathway driven by lipid peroxide accumulation discussed in Section 4.6; that is, α‐tocopherol functions as an endogenous ferroptosis suppressor by trapping lipid radicals independently of the GPX4/glutathione axis, and vitamin E deficiency sensitizes cells to ferroptotic death [125].

Despite this shared antioxidant mechanism, the individual isoforms differ substantially in their anticancer activities. Tocotrienols, particularly γ‐ and δ‐tocotrienol, demonstrate markedly greater growth‐suppressive and proapoptotic effects than tocopherols in breast cancer models [126, 127]. Mechanistically, tocotrienols inhibit NF‐κB, PI3K/Akt, and STAT3 signaling, suppress VEGF‐driven angiogenesis, and induce apoptosis through caspase activation and PARP cleavage in both ER‐positive MCF‐7 and triple‐negative MDA‐MB‐231 cells [128, 129, 130]. The selectivity of tocotrienols for cancer cells over normal mammary epithelial cells has been attributed, at least in part, to their pro‐oxidant activity at pharmacological concentrations: tocotrienols can generate ROS in cancer cells with already elevated oxidative stress, pushing them past the apoptotic threshold while sparing normal cells operating at lower baseline ROS [130, 131]. Additionally, γ‐tocotrienol downregulates *MDR1*‐encoded P‐glycoprotein expression in drug‐resistant breast cancer cells, impairing efflux pump‐mediated chemotherapeutic expulsion and resensitizing resistant cells to treatment (a mechanism with direct implications for the MDR problem discussed in Section 6 below) [132].

Conversely, α‐tocopherol, the most abundant dietary form and the isoform preferentially retained in human tissues, has yielded concerning results in large‐scale chemoprevention trials. The SELECT trial demonstrated that α‐tocopherol supplementation significantly increased prostate cancer incidence, consistent with the antioxidant paradox discussed in the introduction to this section: by reinforcing cancer cell antioxidant defenses, α‐tocopherol may protect incipient tumors from ROS‐mediated elimination [103]. Whether similar risks apply to breast cancer remains unclear, but these findings underscore the importance of distinguishing between isoforms when evaluating vitamin E's therapeutic potential. The absence of large‐scale, survival‐focused clinical trials specifically examining tocotrienols in breast cancer, combined with limited data outside TNBC and highly invasive phenotypes, currently impedes clinical translation. Vitamin E's key characteristics in breast cancer are summarized in Table 1.

### 5.5 Carotenoids

Carotenoids are a class of lipophilic isoprenoid pigments comprising over 700 naturally occurring compounds, of which five—α‐carotene, β‐carotene, lutein/zeaxanthin, lycopene, and β‐cryptoxanthin— account for > 95% of circulating plasma carotenoids and are obtained primarily from dietary fruits and vegetables [152, 153]. The primary antioxidant mechanism of carotenoids is physical quenching of singlet oxygen (^1^O_2_) through energy transfer, a process distinct from the hydrogen atom donation by which vitamins C and E scavenge free radicals. Carotenoids also react with lipid peroxyl radicals, though this activity is oxygen tension‐dependent. At physiological O_2_ levels, carotenoids act as effective chain‐breaking antioxidants, whereas under the low O_2_ tensions characteristic of the tumor microenvironment, carotenoid radical intermediates can become pro‐oxidant, generating oxidative species that damage cancer cell macromolecules [133, 134]. This context‐dependent redox behavior underlies the dual anticancer mechanisms observed in preclinical models: in tumor cells, carotenoids elevate ROS to activate DDRs, induce p53 and p27, suppress PI3K/AKT and NF‐κB survival signaling, and promote apoptosis and cell cycle arrest, while in normal cells they function predominantly as antioxidants [135, 153].

Among specific carotenoids, β‐carotene induces S‐phase cell cycle arrest in TNBC MDA‐MB‐231 cells through JNK pathway modulation [135], and lycopene has demonstrated antiproliferative and pro‐apoptotic effects across multiple breast cancer cell lines, with epidemiological meta‐analyses suggesting an inverse association between dietary lycopene intake and breast cancer risk [136]. Lutein selectively inhibits growth across breast cancer subtypes and enhances the cytotoxic effects of taxane chemotherapeutics through intracellular ROS generation, p53 activation, and HSP60 upregulation [154]. However, clinical translation of carotenoids has been complicated by the results of large‐scale chemoprevention trials. The ATBC trial and the CARET study demonstrated that β‐carotene supplementation significantly increased lung cancer incidence in smokers—a finding consistent with the antioxidant paradox discussed in the introduction to this section, and a cautionary example of how antioxidant supplementation may protect incipient tumor cells from ROS‐mediated elimination under conditions of high oxidative burden [137, 155]. Whether similar risks apply to breast cancer at pharmacological carotenoid doses remains unresolved.

Additional barriers to clinical translation include low aqueous solubility and poor oral bioavailability, which limit achievable plasma concentrations from dietary intake [153]. Encapsulating carotenoids in lipid‐based nanocarriers, polymeric nanoparticles, or nanoemulsions represents a promising strategy to improve tumor delivery and bioavailability [156]. Carotenoids' key biological roles, mechanisms, and therapeutic challenges in breast cancer are summarized in Table 1.

## 6 ROS in Immunotherapy and the Tumor Immune Microenvironment (TME)

While Sections 4 and 5 addressed how ROS drive oncogenic signaling and how antioxidants modulate these processes, the following sections examine how ROS can be therapeutically exploited, first through immunotherapy and the tumor microenvironment, and then through targeted strategies to overcome MDR.

ROS profoundly shape the TME through effects on both innate and adaptive immune cell populations. In the hypoxic tumor interior, elevated ROS stabilize HIF‐1α, driving VEGF‐mediated angiogenesis, extracellular matrix remodeling, and transcriptional programs that promote immunosuppression [157]. HIF‐1α activation recruits and polarizes tumor‐associated macrophages (TAMs) toward the immunosuppressive M2 phenotype, which secretes interleukin‐10 (IL‐10) and transforming growth factor‐beta TGF‐β to support tumor growth and immune evasion. Concurrently, hypoxic cancer cells release chemokines (e.g., CCL26, G‐CSF, and IL‐6) that attract myeloid‐derived suppressor cells (MDSCs), where HIF‐1α induces ENTPD2, a purinergic signaling enzyme that stabilizes MDSCs within the TME. MDSCs in turn suppress antitumor immunity through multiple ROS‐dependent mechanisms: they produce arginase‐1, NO, and ROS that directly impair T‐cell receptor signaling and proliferation; they promote regulatory T cell (Treg) expansion; and they induce T‐cell exhaustion through upregulation of PD‐L1 [157]. Importantly, ROS also directly regulate PD‐L1 expression on tumor cells through NF‐κB‐ and STAT3‐mediated transcriptional activation, creating an additional layer of immune evasion that connects the redox biology discussed in Sections 4.2 and 4.3 to immunotherapy resistance [158].

Beyond immune cells, stromal components of the TME contribute to redox dysregulation. Cancer‐associated fibroblasts (CAFs) generate ROS through NOX4 activity and secrete pro‐oxidant cytokines (IL‐6 and TGF‐β) that sustain NF‐κB signaling in adjacent tumor cells [159]. Hypoxic regions within tumors further complicate the redox landscape: HIF‐1α stabilization under low oxygen tension promotes a metabolic shift toward glycolysis, increases mitochondrial ROS production, and upregulates VEGF‐dependent angiogenesis, all of which modulate therapeutic responsiveness to the ROS‐targeting strategies discussed in Section 6.2.

Recent computational approaches have begun to quantify the relationship between ROS levels and immune cell infiltration in breast cancer. A machine learning‐based assessment of ROS‐related gene expression in TNBC identified distinct prognostic subgroups with differential immune cell compositions, suggesting that ROS profiling could guide immunotherapeutic patient stratification [160]. Similarly, integrative multi‐omics analysis has identified PRDX4 (peroxiredoxin 4) as a redox‐regulated mediator of epithelial‐mesenchymal transition and a predictive marker in metastatic breast cancer, linking the peroxiredoxin antioxidant system (a key Nrf2 target gene product discussed in Section 4.4) to immune evasion and metastatic progression [161].

A central therapeutic paradox emerges from these observations: ROS‐generating therapies such as radiotherapy and anthracycline chemotherapy (e.g., doxorubicin) can induce immunogenic cell death (ICD), characterized by calreticulin surface exposure, ATP release, and HMGB1 secretion that activates dendritic cells and primes antitumor T‐cell responses. However, the same therapies simultaneously elevate oxidative stress throughout the TME, impairing effector T‐cell function and reinforcing immunosuppressive circuits [158, 162]. This paradox explains why immune checkpoint inhibitors (ICIs) targeting PD‐1/PD‐L1 show limited efficacy as monotherapy in breast cancer, with objective response rates of approximately 15% even in selected TNBC cohorts [163], and why combination strategies that manage ROS spatiotemporally are needed. Studies suggest that short‐term systemic antioxidant administration can synergize with immunotherapy by protecting effector T cells, while long‐term antioxidant use may paradoxically enhance tumor progression by shielding cancer cells from immune‐mediated ROS attack [158, 164] (Figure 4).

### 6.1 ROS‐Responsive Nanoagents for Targeted Delivery

The elevated ROS levels characteristic of the breast cancer TME, reported at concentrations 100‐fold or more above those in normal tissues, provide a biochemical basis for tumor‐selective drug delivery [165]. ROS‐responsive nanoagents exploit this differential through incorporation of ROS‐cleavable chemical linkers, principally thioketals, thioethers, and boronate esters, which remain stable in the circulation but undergo selective cleavage in the oxidative tumor environment, enabling controlled payload release at the tumor site [163] (Figure 4).

Several ROS‐responsive platforms have shown promise in preclinical breast cancer models (Table 2). Yang et al. developed polydopamine‐coated thioether‐bond nanoparticles (PsmDE) that matched paclitaxel efficacy in 4T1 breast cancer cells while sparing normal cells, with superior intratumoral drug accumulation compared to non‐responsive nanoparticles [172]. Lu et al. engineered a dual‐responsive nano‐prodrug (BTC) combining a GSH‐activated photosensitizer with a ROS‐cleavable thioketal‐camptothecin conjugate, enabling sequential GSH‐triggered phototherapy followed by ROS‐mediated chemotherapy release, with potent cytotoxicity (IC_50_ = 0.50–0.63 µM) and tumor selectivity in breast cancer models [173]. Xu et al. designed a “tumor nano‐bomb” system (HAIR‐CTF) in which hyaluronic acid‐modified nanoparticles are internalized via hyaluronic acid (HA)‐receptor recognition, disassembled by tumor hyaluronidase, and activated by near‐infrared laser‐triggered ROS surge to release camptothecin and 5‐fluorouridine via thioketal cleavage, simultaneously inducing immunogenic cell death that stimulates antitumor immunity against primary tumors, metastases, and recurrence [174]. Additionally, Banstola et al. developed ROS‐responsive thioketal nanoparticles carrying doxorubicin, the immune stimulator R848, and the chemokine MIP‐3α for coordinated chemo‐immunotherapy in ROS‐rich breast cancer TMEs [175]. Nanoparticle‐mediated delivery of PD‐L1‐targeting agents (e.g., siRNA, BRD4 inhibitors such as JQ1) represents another strategy to overcome immune checkpoint‐mediated resistance in TNBC [163].

**Table 2:** Comparative overview of ROS‐targeted therapeutic strategies evaluated in breast cancer models.

Strategy	Representative agents	ROS mechanism	BC subtype evidence	Development stage	Key advantages	Key limitations	Key Refs.
ETC targeting	Elesclomol, DT‐010	↑ Mitochondrial O_2_^•−^ via ETC disruption; ↓ ATP production	TNBC (4T1), MCF‐7	Phase III (melanoma); preclinical (breast)	Dual effect: ROS elevation + ATP depletion impairs efflux pumps	No breast cancer trials; elesclomol failed phase III primary endpoint (SYMMETRY)	[166, 167, 168]
NOX modulation	Ampelopsin	Disrupts NOX‐derived proliferative ROS signaling; ↓ ERK/NF‐κB	MDA‐MB‐231 (TNBC)	Preclinical	Targets cancer‐specific NOX overexpression	Limited isoform selectivity; normal tissue NOX functions may be compromised	[169, 170]
GSH depletion/GST inhibition	PEITC, DLJ14, BSO	Depletes intracellular GSH; inhibits GST‐mediated drug conjugation	MCF‐7/ADR (resistant), MDA‐MB‐231	Preclinical (BSO reached phase I in other cancers)	Directly disables the major intracellular antioxidant buffer	Systemic GSH depletion risks hepato‐ and cardiotoxicity; BSO associated with cardiac hypertrophy	[166, 171]
Nrf2 inhibition	Brusatol, luteolin, altersolanol B	↓ Nrf2 protein → suppresses ARE‐driven antioxidant gene program (SOD, CAT, HO‐1, NQO1)	MDA‐MB‐231, MCF‐7, BT‐474 (HER2^+^)	Preclinical	Disables master antioxidant transcription program; sensitizes to chemotherapy	Brusatol lacks Nrf2 specificity (global translation inhibitor); normal tissue cytoprotection compromised	[85]
Ferroptosis induction	Erastin (system Xc^−^), RSL3 (GPX4)	↓ GSH via cystine import blockade (erastin); direct GPX4 inhibition (RSL3) → lipid peroxide accumulation	LAR‐TNBC (hypersensitive); mesenchymal‐state cells	Preclinical	Exploits iron/lipid metabolism vulnerability; selective for therapy‐resistant mesenchymal phenotype	Poor PK (erastin: low solubility, rapid clearance); no acceptable therapeutic index in vivo	[12, 44, 100]
ROS‐responsive nanoagents	PsmDE, BTC, HAIR‐CTF, TK‐DOX‐R848	Exploit elevated TME H_2_O_2_ to cleave ROS‐sensitive linkers (TK, thioether, boronate ester) → tumor‐selective payload release	4T1 (TNBC), MCF‐7	Preclinical	Endogenous trigger; tumor‐selective activation; combinable with immunotherapy	Variable intratumoral ROS; off‐target activation in inflamed tissue; GMP scalability unproven	[163, 172, 173, 174, 175]
High‐dose IV vitamin C	Ascorbic acid (IV, mM)	Pro‐oxidant via Fe‐catalyzed autoxidation → extracellular H_2_O_2_	Multiple (non‐subtype‐specific)	Phase I–II (advanced cancers); no breast cancer efficacy RCT	Selective toxicity to cancer cells at pharmacological doses; low normal tissue toxicity	Requires IV administration; oral cannot achieve pro‐oxidant concentrations; no definitive breast cancer RCT	[119, 121, 123]

*Note:* The table summarizes the principal ROS mechanism, breast cancer subtype‐specific evidence, current development stage, and key advantages and limitations for each strategy class. Detailed mechanistic discussions are provided in Sections 5, 6, 7 of the main text.

Abbreviations: ARE, antioxidant response element; BSO, buthionine sulfoximine; ETC, electron transport chain; GMP, good manufacturing practice; GPX4, glutathione peroxidase 4; GSH, glutathione; GST, glutathione S‐transferase; IV, intravenous; LAR, luminal androgen receptor; NOX, NADPH oxidase; PK, pharmacokinetics; RCT, randomized controlled trial; TK, thioketal; TME, tumor microenvironment; TNBC, triple‐negative breast cancer.

ROS‐responsive drug delivery systems offer a distinct advantage over other stimuli‐responsive platforms in that they exploit an endogenous biochemical feature of the tumor microenvironment, requiring no external activation equipment. By contrast, photodynamic/photothermal approaches, including biomimetic nanoreactors that combine photodynamic therapy with glucose oxidase‐mediated starvation to achieve synergistic tumor suppression [176], depend on near‐infrared laser irradiation that is limited by tissue penetration depth and clinical workflow complexity. Similarly, chemiexcited photodynamic systems that generate ROS through intramolecular chemical energy transfer rather than external light [177] address the light‐penetration problem but introduce pharmacokinetic challenges related to the stability and reactivity of chemiluminescent substrates. Multi‐stimuli‐responsive “Boolean logic” platforms, which integrate two or more orthogonal triggers (e.g., acidic pH and reducing environment) to achieve AND‐gate specificity for drug release [178], represent a conceptually elegant strategy for minimizing off‐target activation, but their synthetic complexity and manufacturing scalability remain significant translational barriers. Similarly, binary cooperative prodrug nanoparticles that modulate the immune tumor microenvironment through synergistic chemo‐immunotherapy represent a complementary non‐ROS‐triggered approach to enhancing antitumor efficacy [179].

The principal limitation of ROS‐triggered systems is biological variability: intratumoral H_2_O_2_ concentrations, while consistently elevated relative to normal tissue, vary substantially across breast cancer subtypes, patients, and treatment stages (Section 3), and nonmalignant inflammatory conditions can also produce ROS levels sufficient to trigger premature drug release. Furthermore, the kinetics of ROS‐mediated linker cleavage must be carefully matched to the rate of intracellular drug action to avoid either incomplete release or premature payload degradation. Dual‐ and multi‐responsive designs that combine ROS sensitivity with a second trigger, such as the GSH/ROS sequential activation demonstrated in the BTC system described above, may offer improved tumor selectivity over single‐trigger platforms, although at the cost of increased formulation complexity. Currently, no ROS‐responsive nanoagent has entered clinical trials for any cancer type, and the field requires systematic evaluation of pharmacokinetics, biodistribution, long‐term biocompatibility, and manufacturing reproducibility under (good manufacturing practice) GMP conditions before clinical translation can proceed.

### 6.2 ROS Modulation to Overcome MDR

MDR remains a central obstacle in breast cancer pharmacotherapy, driven by overexpression of drug‐efflux transporters (P‐glycoprotein/MDR1, MRPs), enhanced DNA repair, apoptosis evasion, and upregulation of detoxification systems, many of which are directly regulated by the redox‐sensitive signaling pathways discussed in Sections 4.2, 4.3, 4.4 [166]. Modulating intracellular ROS levels offers a mechanistically rational approach to overcome MDR by exploiting the narrow redox window within which cancer cells operate: strategies that further elevate ROS or disable antioxidant defenses can push resistant cancer cells past the apoptotic threshold while depleting the ATP that powers efflux pumps [166, 180] (Figure 4; Table 2). Recent advances in artificial intelligence have enabled systematic identification of the signaling pathway crosstalk that underlies drug resistance, revealing interconnected ROS‐dependent networks, including PI3K/AKT, NF‐κB, MAPK, and Nrf2 pathways, as convergent nodes that sustain the resistant phenotype. AI‐guided approaches can predict synergistic drug combinations that simultaneously target multiple resistance‐associated pathways, representing a paradigm shift from empirical combination therapy toward computationally informed precision strategies [181]. It should be noted that MDR is a multifactorial phenomenon extending well beyond redox biology to include epigenetic reprogramming, phenotypic plasticity, tumor heterogeneity, and microenvironment‐mediated resistance. This review focuses specifically on redox‐dependent mechanisms of MDR, which intersect with and often potentiate these broader resistance pathways, but do not capture the full complexity of clinical drug resistance.

#### 6.2.1 Targeting the Mitochondrial ETC

Cancer cells with overactive ETC generate elevated ATP that fuels drug‐efflux pumps and elevated ROS against which they maintain adapted antioxidant defenses. Pharmacological ETC disruption simultaneously increases ROS to lethal levels and depletes ATP, thereby weakening both antioxidant buffering and efflux‐mediated resistance [166]. Elesclomol, a clinical‐stage copper ionophore that disrupts mitochondrial ETC function, inhibits breast cancer cell growth as monotherapy and synergizes with doxorubicin and paclitaxel by activating caspase‐3‐dependent apoptosis and suppressing NF‐κB survival signaling [167]. Combined with glycolysis inhibitors (2‐deoxy‐d‐glucose or 3‐bromopyruvate), elesclomol demonstrated enhanced cytotoxicity in MCF‐7 and MDA‐MB‐231 cells, consistent with simultaneous disruption of both oxidative phosphorylation and glycolytic compensation [182]. Additional ETC‐targeting agents with breast cancer activity include rotenoisin A, which selectively induces oxidative stress and MAPK‐mediated apoptosis in MCF‐7 cells while sparing normal cells [168], and DT‐010, which blocks Complex II to reduce ATP and increase ROS in both MCF‐7 and MDA‐MB‐231 cells [166].

#### 6.2.2 Targeting NOX

As discussed in Section 4.2, NOX enzymes are the primary dedicated ROS‐generating enzymes, and their overexpression in breast cancer contributes to genomic instability, proliferative signaling, and drug resistance through sustained activation of the PI3K/AKT and NF‐κB pathways [169]. Paradoxically, pharmacological NOX inhibition reduces the ROS that cancer cells depend on for proliferative signaling while simultaneously disrupting the redox‐dependent survival advantages that sustain resistance. Ampelopsin, a natural flavonoid from *Ampelopsis grossedentata*, inhibits NOX2/4‐derived ROS production, reducing survival and inducing apoptosis in MCF‐7 and MDA‐MB‐231 cells while showing no toxicity to normal breast epithelial cells (MCF‐10A) [170]. The NOX‐PTEN‐PI3K feedforward loop described in Section 4.2 represents an additional therapeutic target: by interrupting NOX‐mediated PTEN oxidation, it may be possible to restore PTEN phosphatase activity and suppress constitutive PI3K/AKT signaling in resistant breast cancer cells.

#### 6.2.3 Targeting the GSH Antioxidant System

Cancer cells, particularly chemotherapy‐resistant populations, rely heavily on elevated GSH levels to neutralize ROS generated by cytotoxic drugs, detoxify drug metabolites via GSH conjugation, and maintain the reducing environment required for DNA repair. High GSH levels have been consistently observed in breast tumors resistant to cisplatin [183] and doxorubicin [184], and GSH levels correlate with treatment outcomes in breast cancer patients [166]. Therapeutic approaches to exploit this dependency include direct GSH depletion, inhibition of GSH biosynthesis, and blockade of glutathione S‐transferases (GSTs) [166].

Phenethyl isothiocyanate (PEITC), a naturally occurring compound from cruciferous vegetables, depletes intracellular GSH and generates ROS that trigger caspase‐9/3‐dependent apoptosis in MDA‐MB‐231 cells [171]. Notably, MCF‐7 cells exhibit resistance to PEITC through high Nrf2 expression and consequent upregulation of GSH biosynthetic enzymes. This mechanism is consistent with the Nrf2‐mediated chemoresistance discussed in section 4.4, while pre‐treatment with N‐acetylcysteine (NAC) reduces Nrf2 levels and restores ROS sensitivity [171]. GSTs, which catalyze GSH conjugation to xenobiotics including chemotherapeutic drugs, are overexpressed in drug‐resistant breast cancers. GST‐O1‐1 activates AKT and ERK signaling to enhance cisplatin resistance [166]. DLJ14, a ligustrazine derivative that inhibits GST activity and reduces GSH levels, significantly enhances doxorubicin accumulation and efficacy in DOX‐resistant MCF‐7/Adm cells and in mouse tumor models [166].

#### 6.2.4 Targeting Nrf2‐Mediated Drug Resistance

The dual role of Nrf2 in cancer—tumor‐suppressive in normal cells but oncogenic when constitutively activated—was discussed in detail in Section 4.4. In the context of drug resistance, constitutively active Nrf2 upregulates a coordinated cytoprotective program encompassing antioxidant enzymes (HO‐1, NQO1, and SOD), GSH biosynthetic machinery (GCLC/GCLM), drug‐metabolizing enzymes, and multidrug resistance transporters (MRPs/ABCCs), collectively enabling cancer cells to detoxify chemotherapeutic drugs and survive oxidative cytotoxicity [81, 185]. Pharmacological inhibition of Nrf2 therefore represents a strategy to simultaneously dismantle multiple resistance mechanisms.

Several Nrf2 inhibitors have demonstrated efficacy in breast cancer models. Altersolanol B induces apoptosis in MCF‐7 cells by activating caspase‐9 and PARP, upregulating Bax, and downregulating Bcl‐2, while suppressing Nrf2 through Keap1/Cul3‐mediated degradation and disrupting AKT/mTOR signaling [85]. Luteolin decreases Nrf2, HO‐1, and Cripto‐1 protein levels in MDA‐MB‐231 cells [85]. Brusatol activates Nrf2 degradation, reduces migration and metastasis, and sensitizes MDA‐MB‐468 and MCF‐7 cells to cisplatin; in combination with trastuzumab, it enhances antitumor activity in HER2‐positive BT‐474 cells [85]. While these preclinical findings are promising, clinical development of Nrf2 inhibitors faces the challenge of achieving cancer cell selectivity without compromising Nrf2‐dependent cytoprotection in normal tissues. Hence, this imposes a therapeutic window that may require tumor‐targeted delivery strategies such as the ROS‐responsive nanoagents discussed in Section 6.1.

#### 6.2.5 Ferroptosis as a Therapeutic Strategy

Ferroptosis, the iron‐dependent form of regulated cell death driven by lipid peroxide accumulation, as introduced in Section 4.6, has emerged as a promising therapeutic avenue in breast cancer, particularly for drug‐resistant and mesenchymal‐phenotype tumors. Cancer cells undergoing epithelial‐to‐mesenchymal transition (EMT), which is a hallmark of metastatic and therapy‐resistant breast cancers, paradoxically acquire heightened sensitivity to ferroptosis due to their dependence on lipid metabolism and altered iron homeostasis [44]. This vulnerability can be therapeutically exploited through multiple strategies: inhibition of the system Xc^−^ cystine/glutamate antiporter (e.g., erastin and sulfasalazine) to deplete intracellular cysteine and GSH; direct inhibition of GPX4, the central lipid peroxide detoxifying enzyme (e.g., RSL3 and ML162); iron‐based approaches that amplify Fenton chemistry‐driven lipid peroxidation; and combination strategies with conventional chemotherapeutics that generate ROS [12, 100].

Recent bioinformatics and machine learning analyses have identified ferroptosis‐related gene signatures with prognostic and immunotherapy‐predictive value in breast cancer. Key ferroptosis regulatory genes and their mechanistic associations with breast cancer outcomes have been validated through integrated computational and experimental approaches [186], while machine learning models have unveiled ferroptosis‐driven regulatory pathways that intersect with immunotherapy responsiveness, suggesting that ferroptosis‐based biomarkers could guide both treatment selection and immunotherapeutic stratification in breast carcinoma [187]. The convergence of ferroptosis induction with immunogenic cell death, through the release of damage‐associated molecular patterns (DAMPs) from ferroptotic cells, further supports the integration of ferroptosis‐targeting strategies with immune checkpoint inhibition in breast cancer [188].

## 7 Future Directions and Conclusions

The evidence reviewed in the preceding sections establishes that ROS are not merely byproducts of aberrant cancer cell metabolism but function as central regulators of breast cancer biology. ROS are implicated in driving oncogenic signaling through PI3K/AKT, MAPK, and NF‐κB pathways (Section 3), enabling immune evasion within the TME (Section 5), and paradoxically creating therapeutic vulnerabilities that can be exploited through redox‐targeted strategies. Several key themes and unresolved questions emerge from this synthesis.

The antioxidant paradox demands clinical resolution. The concentration‐dependent duality of antioxidants (i.e., protective at physiological doses yet potentially tumor‐promoting at pharmacological doses) remains the central translational challenge in this field. Landmark chemoprevention trials (SELECT, ATBC, and CARET) demonstrated that antioxidant supplementation can increase cancer incidence, and recent evidence indicates that antioxidant supplements during breast cancer chemotherapy may be associated with higher recurrence and mortality [189]. In preclinical models, N‐acetylcysteine and vitamin C promoted angiogenesis through the redox‐sensitive transcription factor BACH1 [190]. These findings do not negate the therapeutic potential of pro‐oxidant strategies. High‐dose IV vitamin C, ferroptosis inducers, and ETC‐targeting agents all exploit ROS elevation to selectively kill cancer cells, but the findings underscore that antioxidant interventions in breast cancer must be evaluated in a stratified manner, accounting for tumor subtype, stage, redox phenotype, concurrent therapies, and route of administration [191].

Despite the extensive preclinical evidence reviewed in the preceding sections, the clinical translation of ROS‐modulating strategies in breast cancer remains at an early stage, with a pronounced gap between mechanistic promise and clinical validation. Among the agents discussed, melatonin has the most extensive clinical trial record: multiple randomized controlled trials have evaluated melatonin as an adjuvant during breast cancer chemotherapy, demonstrating significant reductions in cancer‐related fatigue, cognitive impairment, sleep disruption, and depressive symptoms [192, 193, 194]. However, these trials assessed supportive care outcomes rather than direct antitumor efficacy, and no large‐scale randomized controlled trial (RCT) has evaluated melatonin's impact on breast cancer progression or survival.

Similarly, a Phase II randomized trial of curcumin combined with docetaxel in advanced and metastatic breast cancer (*n* = 42) has been completed, but its small sample size limits definitive conclusions [195]. High‐dose intravenous vitamin C has progressed through Phase I–II safety trials in advanced cancers, and a retrospective cohort study reported improved quality of life in breast cancer patients during chemotherapy [196]. However, no breast cancer‐specific efficacy RCT has been completed, and the Phase III VITALITY trial demonstrating efficacy was conducted in colorectal cancer [197]. Elesclomol, the only ETC‐targeting agent discussed here to have entered clinical trials, reached Phase III in melanoma (SYMMETRY trial) in combination with paclitaxel but failed to meet its primary endpoint in the overall population, although a pre‐specified subgroup with normal baseline lactate dehydrogenase showed benefit [198]; no breast cancer trials have been initiated. Critically, none of the ferroptosis inducers (erastin and RSL3), Nrf2 inhibitors (brusatol and luteolin), NOX modulators (ampelopsin), or ROS‐responsive nanoagent platforms discussed in this review have entered clinical trials in any cancer type, remaining entirely in preclinical development (Table 2). This translational gap underscores the need for systematic pharmacokinetic optimization, safety profiling, and biomarker‐guided patient selection before the mechanistic insights reviewed here can be meaningfully tested in breast cancer patients.

A fundamental limitation of all ROS‐modulating therapeutic strategies is that the biochemical targets they exploit—mitochondrial ETC, GSH synthesis, Nrf2 signaling, GPX4 activity—are not cancer‐specific, but are essential for redox homeostasis in normal tissues. The therapeutic rationale rests on the premise that cancer cells operate at higher baseline ROS and therefore have a narrower margin before reaching lethal oxidative thresholds [180], but this margin varies across tissues and patients. Pro‐oxidant approaches carry inherent risks of collateral damage: doxorubicin, the most extensively studied ROS‐generating chemotherapeutic, localizes indiscriminately to mitochondria of cardiomyocytes, where it competes with coenzyme Q_10_ in the ETC to generate superoxide, producing the dose‐limiting cardiotoxicity that remains the primary constraint on anthracycline use [199]. Similarly, cisplatin‐induced nephrotoxicity and ototoxicity are driven in part by off‐target ROS generation in renal tubular and cochlear cells [200].

Strategies that disable antioxidant defenses face analogous challenges. Systemic GSH depletion with buthionine sulfoximine has been associated with cardiac hypertrophy and heart failure in preclinical models [201], and Nrf2 inhibitors such as brusatol lack target specificity: brusatol suppresses global cap‐dependent translation rather than selectively degrading Nrf2, producing off‐target effects and systemic toxicity including hypotension, nausea, and emesis [202, 203]. Because Nrf2 is cytoprotective in hepatocytes, cardiomyocytes, neurons, and immune cells, systemic Nrf2 inhibition could compromise normal tissue defenses against oxidative damage, a concern that has driven interest in tumor‐directed nanoparticle delivery of Nrf2 inhibitors as discussed in Section 6.1. Ferroptosis inducers present additional pharmacokinetic barriers: erastin has poor aqueous solubility and rapid metabolic clearance, limiting its systemic bioavailability, and no ferroptosis‐inducing compound has yet demonstrated an acceptable therapeutic index in vivo [204]. These safety considerations underscore that the transition from preclinical proof‐of‐concept to clinical application will require not only efficacy data but also systematic toxicology studies, careful dose‐finding, and delivery strategies that achieve cancer‐selective ROS modulation while sparing critical normal tissues.

Computational approaches are transforming redox biology from descriptive to predictive. A rapidly emerging frontier is the application of machine learning and multi‐omics integration to identify redox‐related gene signatures with prognostic and therapeutic value in breast cancer. AI‐aided redox signatures (AIARS) constructed from large‐scale transcriptomic data sets have demonstrated robust prognostic performance across independent patient cohorts and can predict immunotherapy and chemotherapy responsiveness [205]. Similar oxidative stress gene signatures built using LASSO regression and Cox modeling have identified novel prognostic biomarkers linked to immune cell infiltration and treatment outcomes [206]. Most recently, integration of bulk and single‐cell RNA sequencing has revealed oxidative stress‐related transcriptional subtypes within breast tumors, uncovering intra‐tumoral redox heterogeneity that bulk‐tissue analyses miss, and identifying subtype‐specific therapeutic vulnerabilities [207]. Together with the AI‐guided identification of signaling crosstalk nodes in drug resistance (Section 6.2), ferroptosis gene signatures predicting immunotherapy response (Section 6.2.5), and PRDX4 as a redox‐EMT biomarker for metastatic disease (Section 6), these computational advances are positioning redox profiling as a clinically actionable tool for patient stratification and treatment selection, potentially enabling a transition from empirical to precision redox oncology.

However, significant methodological and translational barriers must be addressed before redox‐based computational approaches can inform clinical decision‐making. Most existing redox gene signatures have been derived from retrospective transcriptomic data sets (TCGA, GEO, and METABRIC) using supervised machine learning methods such as LASSO regression and random forest classifiers, and their prognostic performance has been validated primarily through internal cross‐validation or testing on a single independent cohort. True external validation across multiple, prospectively collected, clinically annotated data sets remains rare, and the reproducibility of specific gene panels across different patient populations, sequencing platforms, and normalization methods has not been systematically assessed [208]. Overfitting is a particular concern when models are trained on hundreds of redox‐related genes relative to modest sample sizes, and the biological interpretability of purely data‐driven signatures, which may include genes with no established mechanistic link to ROS, remains limited [181]. Furthermore, the clinical utility of redox profiling depends on the development of standardized, clinically deployable assays. Current signatures require bulk RNA sequencing or microarray data, whereas point‐of‐care implementation would demand simpler, targeted platforms such as RT‐qPCR panels or immunohistochemistry‐based surrogates. No redox‐based companion diagnostic has received regulatory approval for any cancer type.

The integration of single‐cell transcriptomics, as demonstrated by Shao et al. [207], offers a path toward resolving intra‐tumoral redox heterogeneity that bulk‐tissue signatures inevitably average out, but single‐cell approaches are currently too costly and computationally intensive for routine clinical deployment. Looking forward, the convergence of redox profiling with spatial transcriptomics and proteomics could enable mapping of ROS gradients within the tumor microenvironment at subcellular resolution, potentially allowing clinicians to predict which tumors will respond to pro‐oxidant strategies, ferroptosis inducers, or Nrf2 inhibitors based on their specific redox architecture — the operational definition of precision redox oncology.

Critical gaps remain in clinical translation. Despite robust preclinical evidence, several barriers impede the clinical application of ROS‐modulating strategies in breast cancer. First, standardized methods for measuring ROS and redox biomarkers in patient samples are lacking; without reproducible quantification, clinical trials cannot reliably stratify patients by redox phenotype. Relatedly, no consensus exists on optimal dosing, timing, or combination regimens for ROS‐modulating agents in breast cancer. The absence of standardized therapeutic protocols reflects both the heterogeneity of the agents involved, ranging from dietary supplements to synthetic small molecules to nanoparticle formulations, and the lack of validated pharmacodynamic biomarkers that could guide dose optimization in individual patients. Second, the interactions between ROS‐modulating agents and immune checkpoint inhibitors are insufficiently characterized. While ROS‐generating therapies can induce immunogenic cell death, they simultaneously impair effector T‐cell function, and the net therapeutic effect of combining these approaches remains unpredictable. Third, ROS‐responsive nanoagents, though promising in preclinical models, are in early development and require systematic evaluation of pharmacokinetics, tumor penetration, and safety in phase I trials. Fourth, Nrf2‐targeting strategies face the fundamental challenge of achieving cancer cell selectivity without compromising cytoprotective Nrf2 functions in normal tissues, a therapeutic window that may require tumor‐directed delivery approaches.

In conclusion, this review highlights that the relationship between ROS and breast cancer is defined by context‐dependent duality at every level: ranging from the opposing effects of moderate versus excessive ROS on cell fate, through the antioxidant versus pro‐oxidant activities of dietary compounds, to the immunostimulatory versus immunosuppressive consequences of oxidative stress in the TME. Therapeutic progress will require moving beyond the binary “antioxidant versus pro‐oxidant” framework toward integrated, subtype‐specific strategies that account for the redox landscape of individual tumors. The convergence of AI‐driven redox profiling, ferroptosis‐based therapeutics, ROS‐responsive drug delivery, and rational combination with immunotherapy offers a path toward precision redox oncology, an approach that harnesses, rather than indiscriminately suppresses, the oxidative biology of breast cancer (Figure 4).

## Author Contributions

Conceptualization: Nikola Hadzi‐Petrushev and Marija Nikolovska. Writing – original draft: Marija Nikolovska and Nikola Hadzi‐Petrushev. Writing – review and editing: Radoslav Stojchevski, Aleksandar Eftimov, Mitko Mladenov, and Dimiter Avtanski. Supervision: Nikola Hadzi‐Petrushev. All authors have read and approved the final manuscript.

## Conflicts of Interest

The authors declare no conflicts of interest.

## Data Availability

Data sharing is not applicable to this article as no data sets were generated or analyzed during the current study.

## References

[1] K. Polyak , “Heterogeneity in Breast Cancer,” Journal of Clinical Investigation 121 (2011): 3786–3788, 10.1172/JCI60534. 21965334 PMC3195489

[2] N. Harbeck and M. Gnant , “Breast Cancer,” Lancet 389, no. 10074 (2017): 1134–1150, 10.1016/S0140-6736(16)31891-8. 27865536

[3] C. Shang and D. Xu , “Epidemiology of Breast Cancer,” Oncologie 24 (2022): 649–663, 10.32604/oncologie.2022.027640.

[4] H. Sung , J. Ferlay , R. L. Siegel , et al., “Global Cancer Statistics 2020: GLOBOCAN Estimates of Incidence and Mortality Worldwide for 36 Cancers in 185 Countries,” CA: A Cancer Journal for Clinicians 71, no. 3 (2021): 209–249, 10.3322/caac.21660. 33538338

[5] M. Arnold , E. Morgan , H. Rumgay , et al., “Current and Future Burden of Breast Cancer: Global Statistics for 2020 and 2040,” Breast 66 (2022): 15–23, 10.1016/j.breast.2022.08.010. 36084384 PMC9465273

[6] H. G. Russnes , O. C. Lingjærde , A. L. Børresen‐Dale , and C. Caldas , “Breast Cancer Molecular Stratification: From Intrinsic Subtypes to Integrative Clusters,” American Journal of Pathology 187 (2017): 2152–2162, 10.1016/j.ajpath.2017.04.022. 28733194

[7] T. Sørlie , “Molecular Portraits of Breast Cancer: Tumour Subtypes as Distinct Disease Entities,” European Journal of Cancer 40, no. 18 (2004): 2667–2675, 10.1016/j.ejca.2004.08.021. 15571950

[8] The Cancer Genome Atlas Network , Comprehensive Molecular Portraits of Human Breast Tumors,” Nature 490, no. 7418 (2012): 61–70, 10.1038/nature11412. 23000897 PMC3465532

[9] A. C. Garrido‐Castro , N. U. Lin , and K. Polyak , “Insights Into Molecular Classifications of Triple‐Negative Breast Cancer: Improving Patient Selection for Treatment,” Cancer Discovery 9 (2019): 176–198, 10.1158/2159-8290.CD-18-1177. 30679171 PMC6387871

[10] F. L. Sarmiento‐Salinas , A. Delgado‐Magallón , J. B. Montes‐Alvarado , et al., “Breast Cancer Subtypes Present a Differential Production of Reactive Oxygen Species (ROS) and Susceptibility to Antioxidant Treatment,” Frontiers in Oncology 9 (2019): 480, 10.3389/fonc.2019.00480. 31231612 PMC6568240

[11] P. Karihtala , P. Auvinen , S. Kauppila , K. M. Haapasaari , A. Jukkola‐Vuorinen , and Y. Soini , “Vimentin, zeb1 and Sip1 Are Up‐Regulated in Triple‐Negative and Basal‐Like Breast Cancers: Association With an Aggressive Tumour Phenotype,” Breast Cancer Research and Treatment 138, no. 1 (2013): 81–90, 10.1007/s10549-013-2442-0. 23412770

[12] C. Gorrini , I. S. Harris , and T. W. Mak , “Modulation of Oxidative Stress as an Anticancer Strategy,” Nature Reviews Drug Discovery 12 (2013): 931–947, 10.1038/nrd4002. 24287781

[13] P. Karihtala , S. Kauppila , Y. Soini , and Arja‐Jukkola‐Vuorinen , “Oxidative Stress and Counteracting Mechanisms in Hormone Receptor Positive, Triple‐Negative and Basal‐Like Breast Carcinomas,” BMC Cancer 11 (2011): 262, 10.1186/1471-2407-11-262. 21693047 PMC3141776

[14] M. Oshi , S. Gandhi , L. Yan , et al., “Abundance of Reactive Oxygen Species (ROS) Is Associated With Tumor Aggressiveness, Immune Response, and Worse Survival in Breast Cancer,” Breast Cancer Research and Treatment 194, no. 2 (2022): 231–241, 10.1007/s10549-022-06633-0. 35639264 PMC9987174

[15] H. Kumar , R. M. Kumar , D. Bhattacharjee , P. Somanna , and V. Jain , “Role of Nrf2 Signaling Cascade in Breast Cancer: Strategies and Treatment,” Frontiers in Pharmacology 13 (2022): 720076, 10.3389/fphar.2022.720076. 35571115 PMC9098811

[16] F. Yang , Y. Xiao , J. H. Ding , et al., “Ferroptosis Heterogeneity in Triple‐Negative Breast Cancer Reveals an Innovative Immunotherapy Combination Strategy,” Cell Metabolism 35, no. 1 (2023): 84–100.e8, 10.1016/j.cmet.2022.09.021. 36257316

[17] S. Reuter , S. C. Gupta , M. M. Chaturvedi , and B. B. Aggarwal , “Oxidative Stress, Inflammation, and Cancer: How Are They Linked?,” Free Radical Biology and Medicine 49 (2010): 1603–1616, 10.1016/j.freeradbiomed.2010.09.006. 20840865 PMC2990475

[18] A. Dubey and B. Sharma , “Targeting Oxidative Stress Biomarkers in Breast Cancer Development and the Potential Protective Effect of Phytochemicals,” Drugs and Drug Candidates 4, no. 2 (2025): 23, 10.3390/ddc4020023.

[19] H. Sies , V. V. Belousov , N. S. Chandel , et al., “Defining Roles of Specific Reactive Oxygen Species (ROS) in Cell Biology and Physiology,” Nature Reviews Molecular Cell Biology 23, no. 7 (2022): 499–515, 10.1038/s41580-022-00456-z. 35190722

[20] P. A. Cerutti , “Prooxidant States and Tumor Promotion,” Science 227, no. 4685 (1985): 375–381, 10.1126/science.2981433. 2981433

[21] J. Jiang , K. Wang , Y. Chen , H. Chen , E. C. Nice , and C. Huang , “Redox Regulation in Tumor Cell Epithelial–Mesenchymal Transition: Molecular Basis and Therapeutic Strategy,” Signal Transduction and Targeted Therapy 2 (2017): 17036, 10.1038/sigtrans.2017.36. 29263924 PMC5661624

[22] J. Li , N. Lin , S. Zhang , C. Chen , L. Weng , and Y. Cao , “The Dual Impact of Oxidative Stress on Breast Cancer,” Scientific Reports 15, no. 1 (2025): 39948, 10.1038/s41598-025-23653-0. 41238690 PMC12618601

[23] H. Greenlee , D. L. Hershman , and J. S. Jacobson , “Use of Antioxidant Supplements During Breast Cancer Treatment: A Comprehensive Review,” Breast Cancer Research and Treatment 115 (2009): 437–452, 10.1007/s10549-008-0193-0. 18839308

[24] D. P. Jones , “Radical‐Free Biology of Oxidative Stress,” American Journal of Physiology – Cell Physiology 295 (2008): C849–C868, 10.1152/ajpcell.00283.2008. 18684987 PMC2575825

[25] H. Sies , C. Berndt , and D. P. Jones , “Oxidative Stress,” Annual Review of Biochemistry 86 (2017): 715–748, 10.1146/annurev-biochem-061516-045037. 28441057

[26] B. Halliwell and J. M. C. Gutteridge , “Free Radicals in Biology and Medicine,” Journal of Free Radicals in Biology & Medicine 1, no. 4 (1985): 331–332. 10.1016/0748-5514(85)90028-5 3939136

[27] J. F. Turrens , “Mitochondrial Formation of Reactive Oxygen Species,” Journal of Physiology 552 (2003): 335–344, 10.1113/jphysiol.2003.049478. 14561818 PMC2343396

[28] M. Valko , D. Leibfritz , J. Moncol , M. T. D. Cronin , M. Mazur , and J. Telser , “Free Radicals and Antioxidants in Normal Physiological Functions and Human Disease,” International Journal of Biochemistry and Cell Biology 39 (2007): 44–84, 10.1016/j.biocel.2006.07.001. 16978905

[29] A. Vermot , I. Petit‐Härtlein , S. M. E. Smith , and F. Fieschi , “Nadph Oxidases (Nox): An Overview From Discovery, Molecular Mechanisms to Physiology and Pathology,” Antioxidants 10, no. 6 (2021): 890, 10.3390/antiox10060890. 34205998 PMC8228183

[30] K. Bedard and K. H. Krause , “The NOX Family of ROS‐Generating Nadph Oxidases: Physiology and Pathophysiology,” Physiological Reviews 87 (2007): 245–313, 10.1152/physrev.00044.2005. 17237347

[31] R. J. Mailloux , “An Update on Methods and Approaches for Interrogating Mitochondrial Reactive Oxygen Species Production,” Redox Biology 45 (2021): 102044, 10.1016/j.redox.2021.102044. 34157640 PMC8220584

[32] J. M. Roscoe and C. S. Sevier , “Pathways for Sensing and Responding to Hydrogen Peroxide at the Endoplasmic Reticulum,” Cells 9 (2020): 2314, 10.3390/cells9102314. 33080949 PMC7603117

[33] C. L. Walker , L. C. D. Pomatto , D. N. Tripathi , and K. J. A. Davies , “Redox Regulation of Homeostasis and Proteostasis in Peroxisomes,” Physiological Reviews 98, no. 1 (2018): 89–115, 10.1152/physrev.00033.2016. 29167332 PMC6335096

[34] M. G. Battelli , L. Polito , M. Bortolotti , and A. Bolognesi , “Xanthine Oxidoreductase in Cancer: More Than a Differentiation Marker,” Cancer Medicine 5 (2016): 546–557, 10.1002/cam4.601. 26687331 PMC4799950

[35] E. L. Cavalieri and E. G. Rogan , “Unbalanced Metabolism of Endogenous Estrogens in the Etiology and Prevention of Human Cancer,” Journal of Steroid Biochemistry and Molecular Biology 125 (2011): 169–180, 10.1016/j.jsbmb.2011.03.008. 21397019 PMC4423478

[36] J. D. Yager and N. E. Davidson , “Estrogen Carcinogenesis in Breast Cancer,” New England Journal of Medicine 354, no. 3 (2006): 270–282, 10.1056/NEJMra050776. 16421368

[37] M. Wei , X. He , N. Liu , and H. Deng , “Role of Reactive Oxygen Species in Ultraviolet‐Induced Photodamage of the Skin,” Cell Division 19 (2024): 1, 10.1186/s13008-024-00107-z. 38217019 PMC10787507

[38] E. I. Azzam , J. P. Jay‐Gerin , and D. Pain , “Ionizing Radiation‐Induced Metabolic Oxidative Stress and Prolonged Cell Injury,” Cancer Letters 327 (2012): 48–60, 10.1016/j.canlet.2011.12.012. 22182453 PMC3980444

[39] H. Liang , X. Zhou , Y. Zhu , et al., “Association of Outdoor Air Pollution, Lifestyle, Genetic Factors With the Risk of Lung Cancer: A Prospective Cohort Study,” Environmental Research 218 (2023): 114996, 10.1016/j.envres.2022.114996. 36481370

[40] H. K. Seitz and F. Stickel , “Molecular Mechanisms of Alcohol‐Mediated Carcinogenesis,” Nature Reviews Cancer 7 (2007): 599–612, 10.1038/nrc2191. 17646865

[41] C. F. Manful , E. Fordjour , D. Subramaniam , A. A. Sey , L. Abbey , and R. Thomas , “Antioxidants and Reactive Oxygen Species: Shaping Human Health and Disease Outcomes,” International Journal of Molecular Sciences 26 (2025): 7520, 10.3390/ijms26157520. 40806653 PMC12347716

[42] E. Maldonado , S. Morales‐Pison , F. Urbina , and A. Solari , “Aging Hallmarks and the Role of Oxidative Stress,” Antioxidants 12 (2023): 651, 10.3390/antiox12030651. 36978899 PMC10044767

[43] Y. Yu , S. Liu , L. Yang , et al., “Roles of Reactive Oxygen Species in Inflammation and Cancer,” MedComm 5 (2024): e519, 10.1002/mco2.519. 38576456 PMC10993368

[44] X. Jiang , B. R. Stockwell , and M. Conrad , “Ferroptosis: Mechanisms, Biology and Role in Disease,” Nature Reviews Molecular Cell Biology 22 (2021): 266–282, 10.1038/s41580-020-00324-8. 33495651 PMC8142022

[45] C. C. Winterbourn , “Reconciling the Chemistry and Biology of Reactive Oxygen Species,” Nature Chemical Biology 4 (2008): 278–286, 10.1038/nchembio.85. 18421291

[46] G. P. Bienert and F. Chaumont , “Aquaporin‐Facilitated Transmembrane Diffusion of Hydrogen Peroxide,” Biochimica et Biophysica Acta – General Subjects 1840 (2014): 1596–1604, 10.1016/j.bbagen.2013.09.017. 24060746

[47] H. Sies and D. P. Jones , “Reactive Oxygen Species (ROS) as Pleiotropic Physiological Signalling Agents,” Nature Reviews Molecular Cell Biology 21 (2020): 363–383, 10.1038/s41580-020-0230-3. 32231263

[48] K. M. Holmström and T. Finkel , “Cellular Mechanisms and Physiological Consequences of Redox‐Dependent Signalling,” Nature Reviews Molecular Cell Biology 15 (2014): 411–421, 10.1038/nrm3801. 24854789

[49] S. S. Sabharwal and P. T. Schumacker , “Mitochondrial ROS in Cancer: Initiators, Amplifiers or an Achilles' Heel?,” Nature Reviews Cancer 14 (2014): 709–721, 10.1038/nrc3803. 25342630 PMC4657553

[50] M. Valko , C. J. Rhodes , J. Moncol , M. Izakovic , and M. Mazur , “Free Radicals, Metals and Antioxidants in Oxidative Stress‐Induced Cancer,” Chemico‐Biological Interactions 160 (2006): 1–40, 10.1016/j.cbi.2005.12.009. 16430879

[51] S. Havermann , C. Büchter , K. Koch , and W. Wätjen , “Role of Oxidative Stress in the Process of Carcinogenesis,” in Studies on Experimental Toxicology and Pharmacology, ed. S. M. Roberts , J. P. Kehrer , and L‐O. Klotz (Humana Press, 2015), 173–198, 10.1007/978-3-319-19096-9_9.

[52] U. S. Srinivas , B. W. Q. Tan , B. A. Vellayappan , and A. D. Jeyasekharan , “ROS and the DNA Damage Response in Cancer,” Redox Biology 25 (2019): 101084, 10.1016/j.redox.2018.101084. 30612957 PMC6859528

[53] Y. Nakabeppu , “Cellular Levels of 8‐Oxoguanine in Either DNA or the Nucleotide Pool Play Pivotal Roles in Carcinogenesis and Survival of Cancer Cells,” International Journal of Molecular Sciences 15 (2014): 12543–12557, 10.3390/ijms150712543. 25029543 PMC4139859

[54] A. J. P. O. De Almeida , J. C. P. L. De Oliveira , L. V. Da Silva Pontes , et al., “ROS: Basic Concepts, Sources, Cellular Signaling, and Its Implications in Aging Pathways,” Oxidative Medicine and Cellular Longevity 2022 (2022): 1225578, 10.1155/2022/1225578. 36312897 PMC9605829

[55] Á. García‐Guede , O. Vera , and I. Ibáñez‐de‐Caceres , “When Oxidative Stress Meets Epigenetics: Implications in Cancer Development,” Antioxidants 9 (2020): 468, 10.3390/antiox9060468. 32492865 PMC7346131

[56] S. Kreuz and W. Fischle , “Oxidative Stress Signaling to Chromatin in Health and Disease,” Epigenomics 8 (2016): 843–862, 10.2217/epi-2016-0002. 27319358 PMC5619053

[57] L. C. Cantley , “The Phosphoinositide 3‐Kinase Pathway,” Science 296, no. 5573 (2002): 1655–1657, 10.1126/science.296.5573.1655. 12040186

[58] T. L. Yuan and L. C. Cantley , “PI3K Pathway Alterations in Cancer: Variations on a Theme,” Oncogene 27 (2008): 5497–5510, 10.1038/onc.2008.245. 18794884 PMC3398461

[59] S. R. Lee , K. S. Yang , J. Kwon , C. Lee , W. Jeong , and S. G. Rhee , “Reversible Inactivation of the Tumor Suppressor PTEN by H_2_O_2_ ,” Journal of Biological Chemistry 277, no. 23 (2002): 20336–20342, 10.1074/jbc.M111899200. 11916965

[60] F. Vazquez , S. R. Grossman , Y. Takahashi , M. V. Rokas , N. Nakamura , and W. R. Sellers , “Phosphorylation of the PTEN Tail Acts as an Inhibitory Switch by Preventing Its Recruitment Into a Protein Complex,” Journal of Biological Chemistry 276, no. 52 (2001): 48627–48630, 10.1074/jbc.C100556200. 11707428

[61] J. Kwon , S. R. Lee , K. S. Yang , et al., “Reversible Oxidation and Inactivation of the Tumor Suppressor PTEN in Cells Stimulated With Peptide Growth Factors,” Proceedings of the National Academy of Sciences of the United States of America 101, no. 47 (2004): 16419–16424, 10.1073/pnas.0407396101. 15534200 PMC534546

[62] N. Koundouros and G. Poulogiannis , “Phosphoinositide 3‐Kinase/Akt Signaling and Redox Metabolism in Cancer,” Frontiers in Oncology 8 (2018): 160, 10.3389/fonc.2018.00160. 29868481 PMC5968394

[63] A. A. Akhiani and A. Martner , “Role of Phosphoinositide 3‐Kinase in Regulation of NOX‐Derived Reactive Oxygen Species in Cancer,” Antioxidants 12 (2023): 67, 10.3390/antiox12010067. PMC9854495 36670929

[64] A. de Vasconcelos e Souza , C. C. de Faria , L. M. Pereira , A. C. F. Ferreira , P. H. M. Torres , and R. S. Fortunato , “Gene Expression and Prognostic Value of NADPH Oxidase Enzymes in Breast Cancer,” International Journal of Molecular Sciences 25, no. 6 (2024): 3464, 10.3390/ijms25063464. 38542437 PMC10970871

[65] S. Pervin , L. Tran , R. Urman , et al., “Oxidative Stress Specifically Downregulates Survivin to Promote Breast Tumour Formation,” British Journal of Cancer 108, no. 4 (2013): 848–858, 10.1038/bjc.2013.40. 23403820 PMC3590675

[66] C. Zhang , T. Lan , J. Hou , et al., “NOX4 Promotes Non‐Small Cell Lung Cancer Cell Proliferation and Metastasis Through Positive Feedback Regulation of PI3K/Akt Signaling,” Oncotarget 5, no. 12 (2014): 4392–4405, 10.18632/oncotarget.2025. 24946933 PMC4147332

[67] A. S. Dhillon , S. Hagan , O. Rath , and W. Kolch , “MAP Kinase Signalling Pathways in Cancer,” Oncogene 26 (2007): 3279–3290, 10.1038/sj.onc.1210421. 17496922

[68] Y. Guo , W. Pan , S. Liu , Z. Shen , Y. Xu , and L. Hu , “ERK/MAPK Signalling Pathway and Tumorigenesis (Review),” Experimental and Therapeutic Medicine 19 (2020): 1997–2007, 10.3892/etm.2020.8454. 32104259 PMC7027163

[69] J. A. McCubrey , M. M. LaHair , and R. A. Franklin , “Reactive Oxygen Species‐Induced Activation of the MAP Kinase Signaling Pathways,” Antioxidants and Redox Signaling 8 (2006): 1775–1789, 10.1089/ars.2006.8.1775. 16987031

[70] J. Heo and S. L. Campbell , “Mechanism of Redox‐Mediated Guanine Nucleotide Exchange on Redox‐Active Rho GTPases,” Journal of Biological Chemistry 280, no. 35 (2005): 31003–31010, 10.1074/jbc.M504768200. 15994296

[71] Y. Son , Y.‐K. Cheong , N.‐H. Kim , H.‐T. Chung , D. G. Kang , and H.‐O. Pae , “Mitogen‐Activated Protein Kinases and Reactive Oxygen Species: How Can ROS Activate MAPK Pathways?,” Journal of Signal Transduction 2011 (2011): 1–6, 10.1155/2011/792639. PMC3100083 21637379

[72] A. Acosta‐Casique , J. B. Montes‐Alvarado , M. Barragán , et al., “ERK Activation Modulates Invasiveness and Reactive Oxygen Species (ROS) Production in Triple Negative Breast Cancer Cell Lines,” Cellular Signalling 101 (2023): 110487, 10.1016/j.cellsig.2022.110487. 36216165

[73] E. F. Wagner and Á. R. Nebreda , “Signal Integration by JNK and p38 MAPK Pathways in Cancer Development,” Nature Reviews Cancer 9 (2009): 537–549, 10.1038/nrc2694. 19629069

[74] R. J. Davis , “Signal Transduction by the JNK Group of MAP Kinases,” Cell 103 (2000): 239–252, 10.1016/s0092-8674(00)00116-1. 11057897

[75] C. Bubici and S. Papa , “JNK Signalling in Cancer: In Need of New, Smarter Therapeutic Targets,” British Journal of Pharmacology 171 (2014): 24–37, 10.1111/bph.12432. 24117156 PMC3874694

[76] Y. T. Ma , C. Li , Y. Shen , et al., “Mechanisms of the JNK/p38 MAPK Signaling Pathway in Drug Resistance in Ovarian Cancer,” Frontiers in Oncology 15 (2025): 1533352, 10.3389/fonc.2025.1533352. 40352594 PMC12063130

[77] M. Yamamoto , T. W. Kensler , and H. Motohashi , “The KEAP1‐NRF2 System: A Thiol‐Based Sensor‐Effector Apparatus for Maintaining Redox Homeostasis,” Physiological Reviews 98 (2018): 1169–1203, 10.1152/physrev.00023.2017. 29717933 PMC9762786

[78] L. Baird and M. Yamamoto , “The Molecular Mechanisms Regulating the KEAP1‐NRF2 Pathway,” Molecular and Cellular Biology 40, no. 13 (2020): e00099‐20, 10.1128/MCB.00099-20. 32284348 PMC7296212

[79] K. I. Tong , Y. Katoh , H. Kusunoki , K. Itoh , T. Tanaka , and M. Yamamoto , “Keap1 Recruits Neh2 Through Binding to ETGE and DLG Motifs: Characterization of the Two‐Site Molecular Recognition Model,” Molecular and Cellular Biology 26, no. 8 (2006): 2887–2900, 10.1128/MCB.26.8.2887-2900.2006. 16581765 PMC1446969

[80] R. Saito , T. Suzuki , K. Hiramoto , et al., “Characterizations of Three Major Cysteine Sensors of Keap1 in Stress Response,” Molecular and Cellular Biology 36, no. 2 (2016): 271–284, 10.1128/MCB.00868-15. 26527616 PMC4719294

[81] M. Rojo de la Vega , E. Chapman , and D. D. Zhang , “NRF2 and the Hallmarks of Cancer,” Cancer Cell 34 (2018): 21–43, 10.1016/j.ccell.2018.03.022. 29731393 PMC6039250

[82] S. Wu , H. Lu , and Y. Bai , “Nrf2 in Cancers: A Double‐Edged Sword,” Cancer Medicine 8 (2019): 2252–2267, 10.1002/cam4.2101. 30929309 PMC6536957

[83] X. J. Wang , Z. Sun , N. F. Villeneuve , et al., “Nrf2 Enhances Resistance of Cancer Cells to Chemotherapeutic Drugs, the Dark Side of Nrf2,” Carcinogenesis 29, no. 6 (2008): 1235–1243, 10.1093/carcin/bgn095. 18413364 PMC3312612

[84] N. Li and X. Zhan , “Machine Learning Identifies Pan‐Cancer Landscape of Nrf2 Oxidative Stress Response Pathway‐Related Genes,” Oxidative Medicine and Cellular Longevity 2022 (2022): 8450087, 10.1155/2022/8450087. 35242279 PMC8886747

[85] S. Ghareghomi , M. Habibi‐Rezaei , M. Arese , L. Saso , and A. A. Moosavi‐Movahedi , “Nrf2 Modulation in Breast Cancer,” Biomedicines 10 (2022): 2668, 10.3390/biomedicines10102668. 36289931 PMC9599257

[86] G. H. Liu , J. Qu , and X. Shen , “NF‐κB/p65 Antagonizes Nrf2‐ARE Pathway by Depriving CBP From Nrf2 and Facilitating Recruitment of HDAC3 to MafK,” Biochimica et Biophysica Acta (BBA) – Molecular Cell Research 1783, no. 5 (2008): 713–727, 10.1016/j.bbamcr.2008.01.002. 18241676

[87] J. D. Wardyn , A. H. Ponsford , and C. M. Sanderson , “Dissecting Molecular Cross‐Talk Between Nrf2 and NF‐κB Response Pathways,” Biochemical Society Transactions 43 (2015): 621–626, 10.1042/BST20150014. 26551702 PMC4613495

[88] D. Wang , D. A. Kreutzer , and J. M. Essigmann , “Mutagenicity and Repair of Oxidative DNA Damage: Insights From Studies Using Defined Lesions,” Mutation Research 400, no. 1–2 (1998): 99–115, 10.1016/s0027-5107(98)00066-9. 9685598

[89] J. Cadet and K. J. A. Davies , “Oxidative DNA Damage & Repair: An Introduction,” Free Radical Biology and Medicine 107 (2017): 2–12, 10.1016/j.freeradbiomed.2017.03.030. 28363603 PMC5510741

[90] R. Huang , H. Chen , J. Liang , et al., “Dual Role of Reactive Oxygen Species and Their Application in Cancer Therapy,” Journal of Cancer 12 (2021): 5543–5561, 10.7150/jca.54699. 34405016 PMC8364652

[91] M. Goldstein and M. B. Kastan , “The DNA Damage Response: Implications for Tumor Responses to Radiation and Chemotherapy,” Annual Review of Medicine 66 (2015): 129–143, 10.1146/annurev-med-081313-121208. 25423595

[92] N. Van Den Tempel , A. N. Zelensky , H. Odijk , et al., “On the Mechanism of Hyperthermia‐Induced BRCA2 Protein Degradation,” Cancers 11, no. 1 (2019): 97, 10.3390/cancers11010097. 30650591 PMC6356811

[93] H. Dutta and N. Jain , “Post‐Translational Modifications and Their Implications in Cancer,” Frontiers in Oncology 13 (2023), 10.3389/fonc.2023.1240115. PMC10546021 37795435

[94] I. Dalle‐Donne , R. Rossi , D. Giustarini , R. Colombo , and A. Milzani , “S‐Glutathionylation in Protein Redox Regulation,” Free Radical Biology and Medicine 43 (2007): 883–898, 10.1016/j.freeradbiomed.2007.06.014. 17697933

[95] C. E. Paulsen and K. S. Carroll , “Cysteine‐Mediated Redox Signaling: Chemistry, Biology, and Tools for Discovery,” Chemical Reviews 113 (2013): 4633–4679, 10.1021/cr300163e. 23514336 PMC4303468

[96] I. Dalle‐Donne , D. Giustarini , R. Colombo , R. Rossi , and A. Milzani , “Protein Carbonylation in Human Diseases,” Trends in Molecular Medicine 9 (2003): 169–176, 10.1016/S1471-4914(03)00031-5. 12727143

[97] B. Liu , Y. Chen , and D. K. St. Clair , “ROS and p53: A Versatile Partnership,” Free Radical Biology and Medicine 44 (2008): 1529–1535, 10.1016/j.freeradbiomed.2008.01.011. 18275858 PMC2359898

[98] A. Ayala , M. F. Muñoz , and S. Argüelles , “Lipid Peroxidation: Production, Metabolism, and Signaling Mechanisms of Malondialdehyde and 4‐Hydroxy‐2‐Nonenal,” Oxidative Medicine and Cellular Longevity 2014 (2014): 1–31, 10.1155/2014/360438. PMC4066722 24999379

[99] J. M. Martínez‐Ramírez , C. Cueto‐Ureña , M. J. Ramírez‐Expósito , and J. M. Martínez‐Martos , “Machine Learning Models Utilizing Oxidative Stress Biomarkers for Breast Cancer Prediction: Efficacy and Limitations in Sentinel Lymph Node Metastasis Detection,” Biomedicines 13, no. 12 (2025): 3107, 10.3390/biomedicines13123107. 41463115 PMC12730245

[100] S. J. Dixon , K. M. Lemberg , M. R. Lamprecht , et al., “Ferroptosis: An Iron‐Dependent Form of Nonapoptotic Cell Death,” Cell 149, no. 5 (2012): 1060–1072, 10.1016/j.cell.2012.03.042. 22632970 PMC3367386

[101] M. Redza‐Dutordoir and D. A. Averill‐Bates , “Activation of Apoptosis Signalling Pathways by Reactive Oxygen Species,” Biochimica et Biophysica Acta – Molecular Cell Research 1863 (2016): 2977–2992, 10.1016/j.bbamcr.2016.09.012. 27646922

[102] N. S. Chandel and D. A. Tuveson , “The Promise and Perils of Antioxidants for Cancer Patients,” New England Journal of Medicine 371, no. 2 (2014): 177–178, 10.1056/NEJMcibr1405701. 25006725

[103] E. A. Klein , I. M. Thompson , C. M. Tangen , et al., “Vitamin E and the Risk of Prostate Cancer: The Selenium and Vitamin E Cancer Prevention Trial (SELECT),” Journal of the American Medical Association 306, no. 14 (2011): 1549, 10.1001/jama.2011.1437. 21990298 PMC4169010

[104] The Alpha‐Tocopherol Beta Carotene Cancer Prevention Study Group , The Effect of Vitamin E and Beta Carotene on the Incidence of Lung Cancer and Other Cancers in Male Smokers,” New England Journal of Medicine 330, no. 15 (1994): 1029–1035, 10.1056/NEJM199404143301501. 8127329

[105] R. J. Reiter , J. C. Mayo , D. X. Tan , R. M. Sainz , M. Alatorre‐Jimenez , and L. Qin , “Melatonin as An Antioxidant: Under Promises but Over Delivers,” Journal of Pineal Research 61 (2016): 253–278, 10.1111/jpi.12360. 27500468

[106] L. C. Manchester , A. Coto‐Montes , J. A. Boga , et al., “Melatonin: An Ancient Molecule That Makes Oxygen Metabolically Tolerable,” Journal of Pineal Research 59 (2015): 403–419, 10.1111/jpi.12267. 26272235

[107] R. J. Reiter , S. Rosales‐Corral , D. X. Tan , M. J. Jou , A. Galano , and B. Xu , “Melatonin as a Mitochondria‐Targeted Antioxidant: One of Evolution's Best Ideas,” Cellular and Molecular Life Sciences 74 (2017): 3863–3881, 10.1007/s00018-017-2609-7. 28864909 PMC11107735

[108] G. Favero , E. Moretti , F. Bonomini , R. J. Reiter , L. F. Rodella , and R. Rezzani , “Promising Antineoplastic Actions of Melatonin,” Frontiers in Pharmacology 9 (2018), 10.3389/fphar.2018.01086. PMC6198052 30386235

[109] E. C. De Arruda Veiga , R. Simões , V. E. Valenti , et al., “Repercussions of Melatonin on the Risk of Breast Cancer: A Systematic Review and Meta‐Analysis,” Revista da Associacao Medica Brasileira 65 (2019), 10.1590/1806-9282.65.5.699. 31166448

[110] S. G. Grant , M. A. Melan , J. J. Latimer , and P. A. Witt‐Enderby , “Melatonin and Breast Cancer: Cellular Mechanisms, Clinical Studies and Future Perspectives,” Expert Reviews in Molecular Medicine 11 (2009): e5, 10.1017/S1462399409000982. 19193248 PMC4301735

[111] S. M. Hill , V. P. Belancio , R. T. Dauchy , et al., “Melatonin: An Inhibitor of Breast Cancer,” Endocrine‐Related Cancer 22 (2015): R183–R204, 10.1530/ERC-15-0030. 25876649 PMC4457700

[112] G. Esposito , F. Bravi , C. Santucci , et al., “Night Shift Work and Breast Cancer Risk in Healthcare Workers: A Systematic Review and Meta‐Analysis,” Occupational Medicine 75, no. 9 (2025): 596–607, 10.1093/occmed/kqaf040. 41525351 PMC12794909

[113] K. Priyadarsini , “The Chemistry of Curcumin: From Extraction to Therapeutic Agent,” Molecules 19 (2014): 20091–20112, 10.3390/molecules191220091. 25470276 PMC6270789

[114] Y. Sun , Q. Li , Y. Huang , et al., “Natural Products for Enhancing the Sensitivity or Decreasing the Adverse Effects of Anticancer Drugs Through Regulating the Redox Balance,” Chinese Medicine (United Kingdom) 19 (2024), 10.1186/s13020-024-00982-2. PMC11334420 39164783

[115] J. Zhu , Q. Li , Z. Wu , Y. Xu , and R. Jiang , “Curcumin for Treating Breast Cancer: A Review of Molecular Mechanisms, Combinations With Anticancer Drugs, and Nanosystems,” Pharmaceutics 16 (2024): 79, 10.3390/pharmaceutics16010079. 38258090 PMC10819793

[116] G. M. Calaf , “Curcumin, Oxidative Stress, and Breast Cancer,” in Cancer: Oxidative Stress and Dietary Antioxidants (2021), 10.1016/B978-0-12-405205-5.00015-5.

[117] W. H. Talib , S. A. Al‐Hadid , M. B. W. Ali , I. H. Al‐Yasari , and M. R. A. Ali , “Role of Curcumin in Regulating p53 in Breast Cancer: An Overview of the Mechanism of Action,” Breast Cancer: Targets and Therapy 10 (2018): 207–217, 10.2147/BCTT.S167812. 30568488 PMC6276637

[118] S. I. Sohn , A. Priya , B. Balasubramaniam , et al., “Biomedical Applications and Bioavailability of Curcumin—An Updated Overview,” Pharmaceutics 13, no. 12 (2021): 2102, 10.3390/pharmaceutics13122102. 34959384 PMC8703330

[119] S. Padayatty and M. Levine , “Vitamin C: The Known and the Unknown and Goldilocks,” Oral Diseases 22 (2016): 463–493, 10.1111/odi.12446. 26808119 PMC4959991

[120] L. Cimmino , I. Dolgalev , Y. Wang , et al., “Restoration of TET2 Function Blocks Aberrant Self‐Renewal and Leukemia Progression,” Cell 170, no. 6 (2017): 1079–1095.e20, 10.1016/j.cell.2017.07.032. 28823558 PMC5755977

[121] Q. Chen , M. G. Espey , A. Y. Sun , et al., “Pharmacologic Doses of Ascorbate Act as a Prooxidant and Decrease Growth of Aggressive Tumor Xenografts in Mice,” Proceedings of the National Academy of Sciences of the United States of America 105, no. 32 (2008): 11105–11109, 10.1073/pnas.0804226105. 18678913 PMC2516281

[122] H. R. Harris , N. Orsini , and A. Wolk , “Vitamin C and Survival Among Women With Breast Cancer: A Meta‐Analysis,” European Journal of Cancer 50, no. 7 (2014): 1223–1231, 10.1016/j.ejca.2014.02.013. 24613622

[123] A. C. Carr and J. Cook , “Intravenous Vitamin C for Cancer Therapy—Identifying the Current Gaps in Our Knowledge,” Frontiers in Physiology 9 (2018), 10.3389/fphys.2018.01182. PMC6115501 30190680

[124] R. Brigelius‐Flohé and M. G. Traber , “Vitamin E: Function and Metabolism,” FASEB Journal 13, no. 10 (1999): 1145–1155. 10385606

[125] E. Mishima , J. Ito , Z. Wu , et al., “A Non‐Canonical Vitamin K Cycle Is a Potent Ferroptosis Suppressor,” Nature 608, no. 7924 (2022): 778–783, 10.1038/s41586-022-05022-3. 35922516 PMC9402432

[126] W. Xu , Y. Mi , P. He , S. He , and L. Niu , “γ‐Tocotrienol Inhibits Proliferation and Induces Apoptosis via the Mitochondrial Pathway in Human Cervical Cancer HeLa Cells,” Molecules 22, no. 8 (2017): 1299, 10.3390/molecules22081299. 28777347 PMC6152108

[127] R. Loganathan , K. R. Selvaduray , K. Nesaretnam , and A. K. Radhakrishnan , “Tocotrienols Promote Apoptosis in Human Breast Cancer Cells by Inducing Poly(ADP‐Ribose) Polymerase Cleavage and Inhibiting Nuclear Factor Kappa‐B Activity,” Cell Proliferation 46, no. 2 (2013): 203–213, 10.1111/cpr.12014. 23510475 PMC6603791

[128] P. W. Sylvester and S. Shah , “Intracellular Mechanisms Mediating Tocotrienol‐Induced Apoptosis in Neoplastic Mammary Epithelial Cells,” Asia Pacific Journal of Clinical Nutrition 14, no. 4 (2005): 366–373. 16326643

[129] V. Aggarwal , D. Kashyap , K. Sak , et al., “Molecular Mechanisms of Action of Tocotrienols in Cancer: Recent Trends and Advancements,” International Journal of Molecular Sciences 20 (2019): 656, 10.3390/ijms20030656. 30717416 PMC6386883

[130] M. S. Haque Ripon , M. Asadul Habib , M. Hossain , et al., “Role of Vitamin E in Prevention of Breast Cancer: An Epidemiological Review,” Asian Journal of Advanced Research and Reports 11 (2020): 37–47, 10.9734/ajarr/2020/v11i330266.

[131] K. Takahashi and G. Loo , “Disruption of Mitochondria During Tocotrienol‐Induced Apoptosis in MDA‐MB‐231 Human Breast Cancer Cells,” Biochemical Pharmacology 67, no. 2 (2004): 315–324, 10.1016/j.bcp.2003.07.015. 14698044

[132] Y. Ding , J. Fan , Z. Fan , and K. Zhang , “γ‐Tocotrienol Reverses Multidrug Resistance of Breast Cancer Cells Through the Regulation of the γ‐Tocotrienol‐NF‐κB‐P‐gp Axis,” Journal of Steroid Biochemistry and Molecular Biology 209 (2021): 105835, 10.1016/j.jsbmb.2021.105835. 33556581

[133] G. W. Burton and K. U. Ingold , “β‐Carotene: An Unusual Type of Lipid Antioxidant,” Science 224, no. 4649 (1984): 569–573, 10.1126/science.6710156. 6710156

[134] A. J. Young and G. M. Lowe , “Antioxidant and Prooxidant Properties of Carotenoids,” Archives of Biochemistry and Biophysics 385, no. 1 (2001): 20–27, 10.1006/abbi.2000.2149. 11361018

[135] A. Baeza‐Morales , M. Medina‐García , P. Martínez‐Peinado , et al., “The Antitumour Mechanisms of Carotenoids: A Comprehensive Review,” Antioxidants 13, no. 9 (2024): 1060, 10.3390/antiox13091060. 39334719 PMC11428676

[136] D. Aune , D. S. Chan , A. R. Vieira , et al., “Dietary Compared With Blood Concentrations of Carotenoids and Breast Cancer Risk: A Systematic Review and Meta‐Analysis of Prospective Studies,” American Journal of Clinical Nutrition 96, no. 2 (2012): 356–363, 10.3945/ajcn.112.034165. 22760559

[137] G. S. Omenn , G. E. Goodman , M. D. Thornquist , et al., “Effects of a Combination of Beta Carotene and Vitamin A on Lung Cancer and Cardiovascular Disease,” New England Journal of Medicine 334, no. 18 (1996): 1150–1155, 10.1056/NEJM199605023341802. 8602180

[138] B. Chen , Y. Zhang , Y. Wang , J. Rao , X. Jiang , and Z. Xu , “Curcumin Inhibits Proliferation of Breast Cancer Cells Through Nrf2‐Mediated Down‐Regulation of Fen1 Expression,” Journal of Steroid Biochemistry and Molecular Biology 143 (2014): 11–18, 10.1016/j.jsbmb.2014.01.009. 24486718

[139] M. Ashrafizadeh , Z. Ahmadi , R. Mohammadinejad , T. Farkhondeh , and S. Samarghandian , “Curcumin Activates the Nrf2 Pathway and Induces Cellular Protection Against Oxidative Injury,” Current Molecular Medicine 20, no. 2 (2019): 116–133, 10.2174/1566524019666191016150757. 31622191

[140] S. Hu , Y. Xu , L. Meng , L. Huang , and H. Sun , “Curcumin Inhibits Proliferation and Promotes Apoptosis of Breast Cancer Cells,” Experimental and Therapeutic Medicine 16, no. 2 (2018): 1266–1272, 10.3892/etm.2018.6345. 30116377 PMC6090267

[141] A. L. Lopresti , “The Problem of Curcumin and Its Bioavailability: Could Its Gastrointestinal Influence Contribute to Its Overall Health‐Enhancing Effects?,” Advances in Nutrition 9 (2018): 41–50, 10.1093/advances/nmx011. 29438458 PMC6333932

[142] D. Shetty , Y. J. Kim , H. Shim , and J. P. Snyder , “Eliminating the Heart From the Curcumin Molecule: Monocarbonyl Curcumin Mimics (MACs),” Molecules 20 (2015): 249–292, 10.3390/molecules20010249. PMC4312668 25547726

[143] J. Moreira , L. Saraiva , M. M. Pinto , and H. Cidade , “Diarylpentanoids With Antitumor Activity: A Critical Review of Structure‐Activity Relationship Studies,” European Journal of Medicinal Chemistry 192 (2020): 112177, 10.1016/j.ejmech.2020.112177. 32172081

[144] R. Stojchevski , S. Velichkovikj , J. Bogdanov , et al., “Assessment of the Anticancer and Antimetastatic Effects of Monocarbonyl Analogs of Curcumin, C66 and B2BrBC, in Breast Cancer Cells,” Cancer Cell International 26 (2026): 90, 10.1186/s12935-026-04184-8. 41566370 PMC12905848

[145] E. Pavlova , R. Stojchevski , N. Hadzi‐Petrushev , J. Bogdanov , M. Mladenov , and D. Avtanski , “The Effect of Curcumin and Its Analogs C66 and B2BrBC on Key Biomarkers of Oxidative Stress in Breast Cancer Cells,” Cell Biochemistry and Biophysics, ahead of print, (2026), 10.1007/s12013-026-02072-6. 41996056

[146] M. Golmohammadi , M. Y. Zamanian , A. M. Al‐Ani , et al., “Targeting STAT3 Signaling Pathway by Curcumin and Its Analogues for Breast Cancer: A Narrative Review,” Animal Models and Experimental Medicine 7, no. 6 (2024): 853–867, 10.1002/ame2.12491. 39219410 PMC11680487

[147] G. P. Nagaraju , S. Aliya , S. F. Zafar , R. Basha , R. Diaz , and B. F. El‐Rayes , “The Impact of Curcumin on Breast Cancer,” Integrative Biology (United Kingdom) 4 (2012), 10.1039/c2ib20088k. 22772921

[148] C. Kuiper and M. C. M. Vissers , “Ascorbate as a Co‐Factor for Fe‐ and 2‐Oxoglutarate Dependent Dioxygenases: Physiological Activity in Tumor Growth and Progression,” Frontiers in Oncology 4 (2014), 10.3389/fonc.2014.00359. PMC4261134 25540771

[149] J. Lin , N. R. Cook , C. Albert , et al., “Vitamins C and E and Beta Carotene Supplementation and Cancer Risk: A Randomized Controlled Trial,” Journal of the National Cancer Institute 101, no. 1 (2009): 14–23, 10.1093/jnci/djn438. 19116389 PMC2615459

[150] L. H. Kushi , R. M. Fee , T. A. Sellers , W. Zheng , and A. R. Folsom , “Intake of Vitamins A, C, and E and Postmenopausal Breast Cancer: The Iowa Women's Health Study,” American Journal of Epidemiology 144, no. 2 (1996): 165–174, 10.1093/oxfordjournals.aje.a008904. 8678048

[151] A. S. Mohd Zaffarin , S. F. Ng , M. H. Ng , H. Hassan , and E. Alias , “Pharmacology and Pharmacokinetics of Vitamin E: Nanoformulations to Enhance Bioavailability,” International Journal of Nanomedicine 15 (2020): 9961–9974, 10.2147/IJN.S276355. 33324057 PMC7733471

[152] M. K. Dehnavi , S. Ebrahimpour‐Koujan , K. Lotfi , and L. Azadbakht , “The Association Between Circulating Carotenoids and Risk of Breast Cancer: A Systematic Review and Dose–Response Meta‐Analysis of Prospective Studies,” Advances in Nutrition 15 (2024): 100135, 10.1016/j.advnut.2023.10.007. 38436219 PMC10694674

[153] L. Koklesova , A. Liskova , M. Samec , et al., “Carotenoids in Cancer Apoptosis—The Road From Bench to Bedside and Back,” Cancers 12 (2020): 2425, 10.3390/cancers12092425. 32859058 PMC7563597

[154] X. Gong , J. Smith , H. Swanson , and L. Rubin , “Carotenoid Lutein Selectively Inhibits Breast Cancer Cell Growth and Potentiates the Effect of Chemotherapeutic Agents Through ROS‐Mediated Mechanisms,” Molecules 23, no. 4 (2018): 905, 10.3390/molecules23040905. 29662002 PMC6017803

[155] J. Blumberg , “The Alpha‐Tocopherol, Beta‐Carotene Cancer Prevention Study in Finland,” Nutrition Reviews 52 (1994): 242–245, 10.1111/j.1753-4887.1994.tb01430.x. 8090376

[156] J. A. Kim , J. H. Jang , and S. Y. Lee , “An Updated Comprehensive Review on Vitamin A and Carotenoids in Breast Cancer: Mechanisms, Genetics, Assessment, Current Evidence, and Future Clinical Implications,” Nutrients 13, no. 9 (2021): 3162, 10.3390/nu13093162. 34579037 PMC8465379

[157] E. N. Mendoza , M. R. Ciriolo , and F. Ciccarone , “Hypoxia‐Induced Reactive Oxygen Species: Their Role in Cancer Resistance and Emerging Therapies to Overcome It,” Antioxidants 14, no. 1 (2025): 94, 10.3390/antiox14010094. 39857427 PMC11762716

[158] A. O'reilly , W. Zhao , S. Wickström , E. S. J. Arnér , and R. Kiessling , “Reactive Oxygen Species: Janus‐Faced Molecules in the Era of Modern Cancer Therapy,” Journal for Immunotherapy of Cancer 12, no. 12 (2024): e009409, 10.1136/jitc-2024-009409. 39645234 PMC11629020

[159] J. S. K. Chan , M. J. Tan , M. K. Sng , et al., “Cancer‐Associated Fibroblasts Enact Field Cancerization by Promoting Extratumoral Oxidative Stress,” Cell Death & Disease 8, no. 1 (2017): e2562, 10.1038/cddis.2016.492. 28102840 PMC5386391

[160] J. Li , Y. Liang , X. Zhao , and C. Wu , “Integrating Machine Learning Algorithms to Systematically Assess Reactive Oxygen Species Levels to Aid Prognosis and Novel Treatments for Triple‐Negative Breast Cancer Patients,” Frontiers in Immunology 14 (2023): 1196054, 10.3389/fimmu.2023.1196054. 37404810 PMC10315494

[161] X. Zhou , L. Xie , J. Liu , G. Liao , and H. Yang , “Integrative Multi‐Omics and Machine Learning Framework Identifies PRDX4 as a Redox‐EMT Regulator and Predictive Marker in Bone‐Metastatic Breast Cancer,” npj Precision Oncology 10 (2026): 43, 10.1038/s41698-025-01240-w. 41571856 PMC12848033

[162] G. Kroemer , L. Galluzzi , O. Kepp , and L. Zitvogel , “Immunogenic Cell Death in Cancer Therapy,” Annual Review of Immunology 31 (2013): 51–72, 10.1146/annurev-immunol-032712-100008. 23157435

[163] R. Wang , X. Huang , X. Chen , and Y. Zhang , “Nanoparticle‐Mediated Immunotherapy in Triple‐Negative Breast Cancer,” ACS Biomaterials Science & Engineering 10, no. 6 (2024): 3568–3598, 10.1021/acsbiomaterials.4c00108. 38815129 PMC11167598

[164] T. Wang and H. Xu , “Multi‐Faced Roles of Reactive Oxygen Species in Anti‐Tumor T Cell Immune Responses and Combination Immunotherapy,” Exploration of Medicine 3 (2022): 77–98, 10.37349/emed.2022.00076.

[165] T. P. Szatrowski and C. F. Nathan , “Production of Large Amounts of Hydrogen Peroxide by Human Tumor Cells,” Cancer Research 51, no. 3 (1991): 794–798. 1846317

[166] Q. Cui , J. Q. Wang , Y. G. Assaraf , et al., “Modulating ROS to Overcome Multidrug Resistance in Cancer,” Drug Resistance Updates 41 (2018): 1–25, 10.1016/j.drup.2018.11.001. 30471641

[167] Y. Qu , J. Wang , M. S. Sim , et al., “Elesclomol, Counteracted by Akt Survival Signaling, Enhances the Apoptotic Effect of Chemotherapy Drugs in Breast Cancer Cells,” Breast Cancer Research and Treatment 121, no. 2 (2010): 311–321, 10.1007/s10549-009-0470-6. 19609669

[168] D. Bak , S. H. Kang , C. Park , B. Y. Chung , and H. W. Bai , “A Novel Radiolytic Rotenone Derivative, Rotenoisin A, Displays Potent Anticarcinogenic Activity in Breast Cancer Cells,” Journal of Radiation Research 62, no. 2 (2021): 249–258, 10.1093/jrr/rrab005. 33615367 PMC7948853

[169] D. e Fathah , S. Ejaz , and Y. Hameed , “Oxidative Stress Induced by Over‐Expressed NADPH Oxidase (NOX) Initiates Breast Cancer, Promotes Metastasis via Epithelial to Mesenchymal Transition (EMT), Remodels Chromatin and Confers Drug Resistance,” Journal of Clinical Breast Cancer (2019), 10.52338/jocbc.2024.4221.

[170] Y. Zhou , F. Shu , X. Liang , et al., “Ampelopsin Induces Cell Growth Inhibition and Apoptosis in Breast Cancer Cells Through Ros Generation and Endoplasmic Reticulum Stress Pathway,” PLoS One 9, no. 2 (2014): e89021, 10.1371/journal.pone.0089021. 24551210 PMC3923868

[171] S. S. Syed Alwi , B. E. Cavell , A. Donlevy , and G. Packham , “Differential Induction of Apoptosis in Human Breast Cancer Cell Lines by Phenethyl Isothiocyanate, a Glutathione Depleting Agent,” Cell Stress and Chaperones 17, no. 5 (2012): 529–538, 10.1007/s12192-012-0329-3. 22351438 PMC3535168

[172] B. Yang , K. Wang , D. Zhang , et al., “Polydopamine‐Modified ROS‐Responsive Prodrug Nanoplatform With Enhanced Stability for Precise Treatment of Breast Cancer,” RSC Advances 9, no. 16 (2019): 9260–9269, 10.1039/c9ra01230c. 35517686 PMC9062053

[173] Z. Lu , G. Xu , X. Yang , et al., “Dual‐Activated Nano‐Prodrug for Chemo‐Photodynamic Combination Therapy of Breast Cancer,” International Journal of Molecular Sciences 23, no. 24 (2022): 15656, 10.3390/ijms232415656. 36555298 PMC9779597

[174] W. Xu , Z. Zeng , Y. Tang , et al., “Spatiotemporal‐Controllable ROS‐Responsive Camptothecin Nano‐Bomb for Chemo/Photo/Immunotherapy in Triple‐Negative Breast Cancer,” Journal of Nanobiotechnology 22, no. 1 (2024): 798, 10.1186/s12951-024-03050-x. 39725974 PMC11673808

[175] A. Banstola , M. Pandit , R. Duwa , J. H. Chang , J. H. Jeong , and S. Yook , “Reactive Oxygen Species‐Responsive Dual‐Targeted Nanosystem Promoted Immunogenic Cell Death Against Breast Cancer,” Bioengineering & Translational Medicine 8, no. 5 (2023): e10379, 10.1002/btm2.10379. 37693071 PMC10487313

[176] Z. Yu , P. Zhou , W. Pan , N. Li , and B. Tang , “A Biomimetic Nanoreactor for Synergistic Chemiexcited Photodynamic Therapy and Starvation Therapy Against Tumor Metastasis,” Nature Communications 9, no. 1 (2018): 5044, 10.1038/s41467-018-07197-8. PMC6262009 30487569

[177] Q. Wang , C. Zhang , Y. Zhao , et al., “Polyprodrug Nanomedicine for Chemiexcitation‐Triggered Self‐Augmented Cancer Chemotherapy and Gas Therapy,” Biomaterials 309 (2024): 122606, 10.1016/j.biomaterials.2024.122606. 38776593

[178] B. Hou , L. Zhou , H. Wang , et al., “Engineering Stimuli‐Activatable Boolean Logic Prodrug Nanoparticles for Combination Cancer Immunotherapy,” Advanced Materials 32, no. 12 (2020): 1907210, 10.1002/adma.201907210. 32048361

[179] B. Feng , F. Zhou , B. Hou , et al., “Binary Cooperative Prodrug Nanoparticles Improve Immunotherapy by Synergistically Modulating Immune Tumor Microenvironment,” Advanced Materials 30, no. 38 (2018): 1803001, 10.1002/adma.201803001. 30063262

[180] D. Trachootham , J. Alexandre , and P. Huang , “Targeting Cancer Cells by ROS‐Mediated Mechanisms: A Radical Therapeutic Approach?,” Nature Reviews Drug Discovery 8 (2009): 579–591, 10.1038/nrd2803. 19478820

[181] E. Sutanto , R. Sutanto , S. Velichkovikj , et al., “AI‐Guided Discovery of Oncogenic Signaling Crosstalk in Tumor Progression and Drug Resistance,” Oncology Research 34 (2026): 4, 10.32604/or.2026.076157. PMC13126398 42065041

[182] J. S. Modica‐Napolitano , M. Murray , J. Thibault , et al., “The In Vitro Cytotoxic Effect of Elesclomol on Breast Adenocarcinoma Cells Is Enhanced by Concurrent Treatment With Glycolytic Inhibitors,” Cancers 16, no. 23 (2024): 4054, 10.3390/cancers16234054. 39682240 PMC11639995

[183] S. U. Khan , K. Fatima , S. Aisha , and F. Malik , “Unveiling the Mechanisms and Challenges of Cancer Drug Resistance,” Cell Communication and Signaling 22 (2024): 109, 10.1186/s12964-023-01302-1. 38347575 PMC10860306

[184] N. Traverso , R. Ricciarelli , M. Nitti , et al., “Role of Glutathione in Cancer Progression and Chemoresistance,” Oxidative Medicine and Cellular Longevity 2013 (2013): 1–10, 10.1155/2013/972913. PMC3673338 23766865

[185] C. Glorieux , C. Enríquez , C. González , G. Aguirre‐Martínez , and P. Buc Calderon , “The Multifaceted Roles of NRF2 in Cancer: Friend or Foe?,” Antioxidants 13 (2024): 70, 10.3390/antiox13010070. 38247494 PMC10812565

[186] S. Liang , Y. M. Bai , and B. Zhou , “Identification of Key Ferroptosis Genes and Mechanisms Associated With Breast Cancer Using Bioinformatics, Machine Learning, and Experimental Validation,” Aging 16, no. 2 (2024): 1781–1795, 10.18632/aging.205459. 38244591 PMC10866432

[187] L. Xia , Z. Ye , M. Zheng , and Z. Tan , “Synergistic Bioinformatics and Sophisticated Machine Learning Unveil Ferroptosis‐Driven Regulatory Pathways and Immunotherapy Potential in Breast Carcinoma,” Discover Oncology 16, no. 1 (2025): 668, 10.1007/s12672-025-02393-7. 40320501 PMC12050258

[188] D. Tang , X. Chen , R. Kang , and G. Kroemer , “Ferroptosis: Molecular Mechanisms and Health Implications,” Cell Research 31 (2021): 107–125, 10.1038/s41422-020-00441-1. 33268902 PMC8026611

[189] C. B. Ambrosone , G. R. Zirpoli , A. D. Hutson , et al., “Dietary Supplement Use During Chemotherapy and Survival Outcomes of Patients With Breast Cancer Enrolled in a Cooperative Group Clinical Trial (SWOG S0221),” Journal of Clinical Oncology 38, no. 8 (2020): 804–814, 10.1200/JCO.19.01203. 31855498 PMC7062457

[190] T. Wang , Y. Dong , Z. Huang , et al., “Antioxidants Stimulate BACH1‐Dependent Tumor Angiogenesis,” Journal of Clinical Investigation 133 20 (2023): e169671, 10.1172/JCI169671. 37651203 PMC10575724

[191] I. I. C. Chio and D. A. Tuveson , “ROS in Cancer: The Burning Question,” Trends in Molecular Medicine 23 (2017): 411–429, 10.1016/j.molmed.2017.03.004. 28427863 PMC5462452

[192] A. C. S. Palmer , M. Zortea , A. Souza , et al., “Clinical Impact of Melatonin on Breast Cancer Patients Undergoing Chemotherapy; Effects on Cognition, Sleep and Depressive Symptoms: A Randomized, Double‐Blind, Placebo‐Controlled Trial,” PLoS One 15, no. 4 (2020): e0231379, 10.1371/journal.pone.0231379. 32302347 PMC7164654

[193] A. Sedighi Pashaki , F. Sheida , L. Moaddab Shoar , et al., “A Randomized, Controlled, Parallel‐Group, Trial on the Long‐Term Effects of Melatonin on Fatigue Associated With Breast Cancer and Its Adjuvant Treatments,” Integrative Cancer Therapies 22 (2023): 1–10, 10.1177/15347354231168624. PMC10161337 37139718

[194] F. Nimee , A. Gioxari , P. Papandreou , et al., “The Effect of Melatonin Supplementation on Cancer‐Related Fatigue During Chemotherapy Treatment of Breast Cancer Patients: A Double‐Blind, Randomized Controlled Study,” Cancers 16, no. 4 (2024): 802, 10.3390/cancers16040802. 38398193 PMC10887218

[195] P. J. Judith , B. Maureen , P. Mélanie , et al., “Curcumin's Effect in Advanced and Metastatic Breast Cancer Patients Treated With First or Second‐Line Docetaxel: A Randomized Trial,” Health Science Reports 7, no. 9 (2024): 70052, 10.1002/hsr2.70052. PMC11403303 39286738

[196] C. Vollbracht , B. Schneider , V. Leendert , G. Weiss , L. Auerbach , and J. Beuth , “Intravenous Vitamin C Administration Improves Quality of Life in Breast Cancer Patients During Chemo‐/Radiotherapy and Aftercare: Results of a Retrospective, Multicentre, Epidemiological Cohort Study in Germany,” In Vivo (Athens, Greece) 25, no. 6 (2011): 983–990. 22021693

[197] F. Wang , M. M. He , J. Xiao , et al., “A Randomized, Open‐Label, Multicenter, Phase 3 Study of High‐Dose Vitamin C Plus FOLFOX ± Bevacizumab versus FOLFOX ± Bevacizumab in Unresectable Untreated Metastatic Colorectal Cancer (VITALITY Study),” Clinical Cancer Research 28, no. 19 (2022): 4232–4239, 10.1158/1078-0432.CCR-22-0655. 35929990 PMC9527503

[198] S. J. O'Day , A. M. M. Eggermont , V. Chiarion‐Sileni , et al., “Final Results of Phase III Symmetry Study: Randomized, Double‐Blind Trial of Elesclomol Plus Paclitaxel Versus Paclitaxel Alone as Treatment for Chemotherapy‐Naive Patients With Advanced Melanoma,” Journal of Clinical Oncology 31, no. 9 (2013): 1211–1218, 10.1200/JCO.2012.44.5585. 23401447

[199] E. Panieri and M. M. Santoro , “ROS Homeostasis and Metabolism: A Dangerous Liason in Cancer Cells,” Cell Death and Disease 7 (2016): e2253, 10.1038/cddis.2016.105. 27277675 PMC5143371

[200] T. Karasawa and P. S. Steyger , “An Integrated View of Cisplatin‐Induced Nephrotoxicity and Ototoxicity,” Toxicology Letters 237 (2015): 219–227, 10.1016/j.toxlet.2015.06.012. 26101797 PMC4516600

[201] S. J. Kim , H. S. Kim , and Y. R. Seo , “Understanding of ROS‐Inducing Strategy in Anticancer Therapy,” Oxidative Medicine and Cellular Longevity 2019 (2019): 1–12, 10.1155/2019/5381692. PMC6939418 31929855

[202] S. J. Cai , Y. Liu , S. Han , and C. Yang , “Brusatol, an NRF2 Inhibitor for Future Cancer Therapeutic,” Cell & Bioscience 9, no. 1 (2019): 45, 10.1186/s13578-019-0309-8. 31183074 PMC6554866

[203] A. Olayanju , I. M. Copple , H. K. Bryan , et al., “Brusatol Provokes a Rapid and Transient Inhibition of Nrf2 Signaling and Sensitizes Mammalian Cells to Chemical Toxicity—Implications for Therapeutic Targeting of Nrf2,” Free Radical Biology and Medicine 78 (2015): 202–212, 10.1016/j.freeradbiomed.2014.11.003. 25445704 PMC4291150

[204] X. Chen , R. Kang , G. Kroemer , and D. Tang , “Broadening Horizons: The Role of Ferroptosis in Cancer,” Nature Reviews Clinical Oncology 18 (2021): 280–296, 10.1038/s41571-020-00462-0. 33514910

[205] T. Wang , S. Wang , Z. Li , J. Xie , K. Du , and J. Hou , “Machine Learning Unveils Key Redox Signatures for Enhanced Breast Cancer Therapy,” Cancer Cell International 24, no. 1 (2024): 368, 10.1186/s12935-024-03534-8. 39522039 PMC11549853

[206] D. Hu , B. Qin , L. Zhang , and H. Bu , “Construction of an Oxidative Stress‐Associated Genes Signature in Breast Cancer by Machine Learning Algorithms,” Journal of International Medical Research 52, no. 3 (2024): 1–17, 10.1177/03000605241232560. PMC10960342 38520254

[207] Y. Shao , Y. Zhang , J. Chen , et al., “Comprehensive Analysis of Breast Cancer Oxidative Stress Related Gene Signature: A Combination of Bulk and Single‐Cell RNA Sequencing Analysis,” Mammalian Genome 36, no. 2 (2025): 692–707, 10.1007/s00335-025-10130-2. 40274661

[208] O. Pich , C. Bailey , T. B. K. Watkins , S. Zaccaria , M. Jamal‐Hanjani , and C. Swanton , “The Translational Challenges of Precision Oncology,” Cancer Cell 40 (2022): 458–478, 10.1016/j.ccell.2022.04.002. 35487215

